# Single-Step Upcycling of Sugarcane Bagasse and Iron Scrap into Magnetic Carbon for High-Performance Adsorbents

**DOI:** 10.3390/molecules30092040

**Published:** 2025-05-03

**Authors:** Sirinad Mahawong, Piyatida Thaveemas, Parichart Onsri, Sulawan Kaowphong, Waralee Watcharin, Supanna Techasakul, Decha Dechtrirat, Laemthong Chuenchom

**Affiliations:** 1 Division of Physical Science, Faculty of Science, Prince of Songkla University, Songkhla 90112, Thailand; sirinadmahawong@gmail.com; 2 Laboratory of Organic Synthesis, Chulabhorn Research Institute, Bangkok 10210, Thailand; piyatidat@cri.or.th (P.T.); paricharto@cri.or.th (P.O.); supanna@cri.or.th (S.T.); 3 Department of Chemistry, Center of Excellence in Materials Science and Technology, Faculty of Science, Chiang Mai University, Chiang Mai 50200, Thailand; sulawank@gmail.com; 4 Faculty of Biotechnology, Assumption University, Hua Mak Campus, Bangkok 10240, Thailand; waraleewtc@au.edu; 5 Department of Materials Science, Faculty of Science, Kasetsart University, Bangkok 10900, Thailand; 6 Specialized Center of Rubber and Polymer Materials for Agriculture and Industry (RPM), Faculty of Science, Kasetsart University, Bangkok 10900, Thailand; 7 Center of Excellence for Innovation in Chemistry, Faculty of Science, Prince of Songkla University, Songkhla 90112, Thailand

**Keywords:** biomass, iron scrap, carbonization, magnetization, activation, adsorption, valorization

## Abstract

The sugar industry produces significant quantities of waste biomass, while other industrial sectors generate iron scrap as waste. This study seeks to make use of these waste products using an in situ approach that integrates carbonization, activation, and magnetization to convert sugarcane waste and iron scrap into a magnetic carbon composite adsorbent. The porosity of the activated carbon was enhanced by the activating agent potassium hydroxide (KOH) and further improved by the addition of iron scrap, which also imparted magnetic properties to the composite. The developed porosity of the composite increased the overall adsorption capacity of the adsorbent. The synthesis conditions were varied to examine the effects on the properties of the adsorbent. The amount of KOH used in the synthesis influenced the performance of the material. The best-performing adsorbent demonstrated strong potential in the treatment of wastewater by exhibiting an adsorption capacity of 1736.93 mg/g for the antibiotic tetracycline. The magnetic properties of the composite adsorbent enable simple separation and recovery, making the adsorbent reusable and lowering operating costs. This study provides a clear framework for the synthesis of waste-derived magnetic carbon composite adsorbents that can offer financial and environmental advantages while remaining effective in industrial contexts.

## 1. Introduction

The industrial, agricultural, and domestic discharge of chemical pollutants into natural water supplies has become a significant environmental concern due to the health risks posed by these contaminants [1]. Notably, antibiotics present a significant threat to the environment and public health. Tetracycline (TC) is a common antibiotic used for both human and veterinary purposes. It is often found in agricultural runoff, as it is used in livestock production [2]. When released into aquatic ecosystems, it tends to accumulate, causing harm to aquatic organisms and further exacerbating the global problem of antibiotic resistance [3]. TC contamination, therefore, poses a threat to biodiversity as well as human health, and for these reasons, it is necessary to find a sustainable means of addressing this problem. Numerous approaches have been employed to eliminate TC antibiotics, such as oxidation [4], coagulation and flocculation [5], electrodegradation [6], photocatalytic degradation [7], ozonation [8], biodegradation [9], and adsorption [10,11,12,13,14,15]. Adsorption-based methods tend to be among the more effective techniques since they are relatively inexpensive and straightforward to implement [16,17].

In the removal of antibiotics, carbonaceous materials have proven effective. Activated carbon [18], carbon aerogel [19], carbon nanotubes (CNTs) [20], and graphene sheets along with their oxides [21] are highly versatile. They offer good physical and chemical properties along with a large surface area and a high degree of porosity, providing numerous interactive sites that can trap pollutants. They also exhibit strong physical, chemical, and thermal durability, allowing them to be used over long periods in treating water, even when exposed to highly acidic or alkaline conditions or excessive heat. Activated carbon is typically found in the form of fine powders [22]; however, the small particle size makes collection after use difficult. As a result, recycling becomes problematic, potentially causing secondary environmental contamination and rendering large-scale applications impractical. Magnetic carbon adsorbents are therefore attracting significant interest as an alternative material for water treatment since they can be collected and reused after pollutant adsorption simply by using a magnet [23]. This quick and efficient process reduces costs, accelerates the water treatment process, enables reuse of the adsorbent, and makes this approach feasible on a large scale. 

Despite these advancements, a clear research gap remains in developing a low-cost, sustainable, and scalable method for producing magnetic carbon adsorbents directly from waste materials. To date, no study has reported the use of iron scrap and sugarcane bagasse—two abundant industrial and agricultural wastes—in a one-step synthesis approach that simultaneously integrates carbonization, activation, and magnetization. This study uniquely addresses that gap by providing a novel and eco-friendly strategy for tetracycline removal using waste-derived precursors in a simplified, energy-efficient process.

While magnetic carbon adsorbents offer significant promise, the techniques commonly employed to magnetize carbon-based materials typically make use of multi-step procedures with separate carbonization, activation, and magnetization stages [24,25,26], although in some cases these stages occur in pairs, followed by the third stage; for instance, carbonization and activation take place together, followed by magnetization [27,28,29,30]. Alternatively, the first stage can occur in isolation, followed by the other two stages together, such as carbonization initially, followed by magnetization and activation together [31,32]. In large-scale operations, multi-step synthesis procedures are often slow, expensive, and operationally complicated, as each additional step adds to the overall consumption of materials, energy, labor, and time. For instance, conventional approaches require separate heating cycles for carbonization and activation, followed by another distinct step for magnetization—each demanding specific conditions, equipment, and post-processing efforts. These segmented stages not only increase energy and chemical usage but also introduce opportunities for material loss and inconsistencies in product quality. In contrast, the in situ integration of carbonization, activation, and magnetization into a single-step process offers a streamlined and highly efficient alternative. By combining all three transformations in one thermal treatment, porous magnetic carbon can be synthesized more quickly and with substantially lower energy input [33,34,35]. Furthermore, eliminating separate processing stages reduces the number of chemical washing steps and material handling operations, thereby minimizing waste generation and operational costs. Additionally, by requiring only one heating setup and fewer raw materials, the integrated method enhances scalability and reproducibility, making it particularly attractive for industrial-scale production of magnetic biochar or carbon-based adsorbents.

The principal sources of magnetism involved in the synthesis of magnetic carbon are iron salts such as FeCl_3_, FeSO_4_, and Fe(NO_3_)_3_ [36,37,38], but these compounds can be costly. The use of a cheaper alternative would thus lower the cost of synthesizing magnetic carbon adsorbents. Underutilized iron-rich waste products, such as iron scrap, offer significant potential due to their availability and low cost, while their application provides additional environmental benefits via the recycling and reuse of this industrial waste.

The source of carbon is also a major consideration for the large-scale production of magnetic carbon materials. Globally, one of the largest agricultural waste products in terms of quantity is sugarcane bagasse. Dry sugarcane bagasse is produced in large amounts during sugar extraction from sugarcane, and much of it is burned in the field, contributing to air pollution [39]. While this practice is environmentally harmful, sugarcane bagasse has nevertheless shown potential for beneficial processing [40]. It is cheap, is readily available, and contains high proportions of cellulose, hemicellulose, and lignin. Therefore, its application as a carbon source offers a way to dispose of sugarcane bagasse in a beneficial manner, producing large quantities of low-cost, carbon-based material that can be used as an adsorbent for wastewater treatment [41]. In addition to its low commercial value and high carbon content, sugarcane bagasse offers several advantages over other biomass sources. Its low ash content [42] helps reduce the formation of non-carbon residues that could affect pore structure and adsorption efficiency [43]. Its fibrous, porous structure enhances pore formation, yielding activated carbon with a well-developed pore network [44]. Additionally, as an annually harvested crop, sugarcane provides a sustainable and renewable material supply [45].

This study, therefore, presents a sustainable and integrated approach for producing magnetic carbon adsorbents from waste iron scrap and sugarcane bagasse in a single-step process. By combining carbonization, activation, and magnetization in one operation, the method reduces material usage and energy consumption, shortens processing time, and eliminates the need for chemical iron salts. The proposed strategy not only offers a cost-effective solution for antibiotic removal but also promotes circular economy principles through the valorization of industrial and agricultural wastes.

## 2. Results and Discussion

### 2.1. Material Characterization

The reaction between iron scrap and hydrochloric acid generated soluble Fe^2+^ and Fe^3+^ ions. The ions formed bonds with electron-rich oxygen atoms of the polar hydroxyl and ether groups of cellulose, which anchored the iron ions to the surface and pores of the bagasse [46]. When the precursor solution was mixed with the KOH solution, KOH impregnated the carbon matrix. When the dried sample was thermally treated by pyrolysis, activation of the carbon matrix by KOH during pyrolysis generated a highly porous structure, which increased the surface area of the material. This process is also observed in conventionally produced activated carbon [47]. At the same time, the iron ions on and within the carbon matrix served as precursors for the formation of magnetic particles throughout the composite. The resulting material was labeled MCC-x, where MCC refers to the magnetic carbon composite and x denotes the KOH-to-biomass ratio used during synthesis.

**Figure 1:**
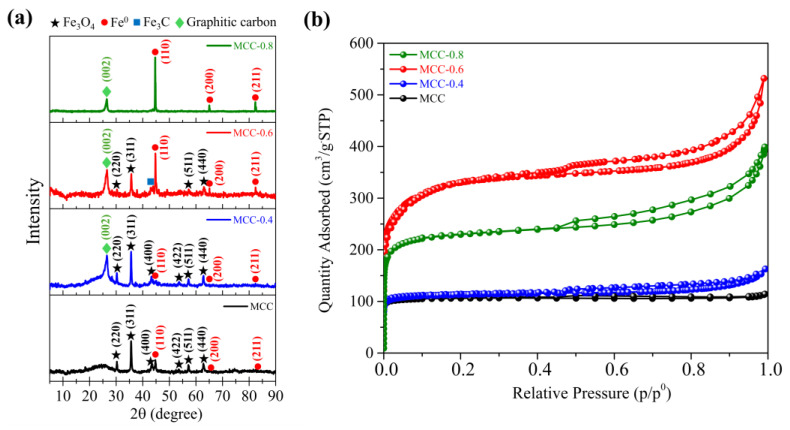
XRD patterns (**a**) and nitrogen adsorption–desorption isotherms of MCC, MCC-0.4, MCC-0.6, and MCC-0.8 (**b**).

The crystallography and iron phases of the various adsorbents were examined using an XRD analysis. The XRD patterns revealed the formation of iron and iron oxide (Figure 1a). Without KOH, the MCC sample primarily exhibited magnetite (Fe_3_O_4_), as confirmed by characteristic peaks observed at 30.13°, 35.48°, 43.12°, 53.50°, 57.03°, and 62.63° 2θ, which correspond to the (220), (311), (400), (422), (511), and (440) planes of Fe_3_O_4_, respectively (JCPDS No. 01-075-0033). Furthermore, the peaks at 44.60°, 64.68°, and 82.35° 2θ, corresponding to the (110), (200), and (211) planes of Fe^0^, indicate the coexistence of metallic iron in the sample (JCPDS No. 00-001-1262). The detection of Fe_3_O_4_ and Fe^0^ implies that non-magnetic Fe^2+^ and Fe^3+^ ions were converted into magnetic phases of Fe_3_O_4_ and Fe^0^ during pyrolysis. 

The mechanisms by which magnetite and metallic iron are formed are complex but primarily depend on temperature [36,48,49,50]. When iron scrap was dissolved in hydrochloric acid, Fe^2+^ and Fe^3+^ ions were released and subsequently hydrolyzed to produce a variety of iron hydroxyl species. Upon dehydration, these species were transformed at higher temperatures into goethite. The goethite was subsequently dehydrated to form maghemite when the temperature exceeded 220 °C. With continued heating, the structure was rearranged so that maghemite became hematite. A further rise in temperature beyond 560 °C triggered a reaction between hematite and carbon, resulting in the formation of magnetite. As the temperature surpassed 700 °C, iron oxide species such as hematite and magnetite underwent reduction to zero-valent Fe^0^ [50], which in turn led to further changes in the carbon structure.

When KOH was introduced in increasing quantities, a transition of the iron phase from Fe_3_O_4_ to Fe^0^ was observed. This was attributed to the reaction of KOH with carbon in the feedstock, which took place when the temperature exceeded 700 °C and produced H_2_ gas—a powerful reducing agent [47]. As the H_2_ concentration increased, the reducing environment was further strengthened, thereby facilitating the transformation of iron [51]. As a result, MCC-0.8 exhibited only the Fe^0^ phase. Moreover, a peak at 26.23°, corresponding to the (002) plane of graphitic carbon (JCPDS No. 01-075-1621), was observed in the XRD patterns of all the samples that had been exposed to KOH. This outcome may be due to the formation of Fe_3_C in the reducing atmosphere. Fe_3_C can be formed as an intermediate during the course of high-temperature pyrolysis or carbothermal reduction due to the reaction between iron and carbon. Fe_3_C may act as a catalyst, promoting the reorganization of carbon atoms to produce ordered graphitic structures [52,53,54,55]. 

KOH is often employed to activate pores in carbon materials. The proposed mechanism [47] is described by Equations R1–R4 (Note S1). Before activation begins, the carbon precursor is impregnated with KOH. As the temperature rises during pyrolysis, though still below 700 °C, the carbon structure is broken down by KOH to produce K_2_CO_3_. The thermal decomposition of K_2_CO_3_ produces K_2_O along with CO_2_ gas. The resulting CO_2_ serves as a gasifying agent, leading to the formation of pores in the carbon material. Once the temperature exceeds 700 °C, K_2_CO_3_ and K_2_O react further with carbon to generate CO. As carbon is consumed in the release of this gas, additional pores are formed. In the activation stage, etching of the carbon structure leads to the formation of micropores, which subsequently expand to mesopores [56,57]. Without KOH (MCC), pores still develop during the carbothermal process as carbon reacts at high temperatures with Fe_2_O_3_ or Fe_3_O_4_ to produce metallic iron and CO_2_. The released CO_2_ generates pores in the carbon material and continues to act as a gasifying agent, reacting further with carbon and etching the matrix to create additional pores [50]. 

To verify the development of porosity and determine how KOH affected it, a N_2_ sorption analysis was performed. Based on the IUPAC classification, the N_2_ sorption isotherms of all samples were mixed type I/type IV isotherms (Figure 1b). In a type I N_2_ sorption isotherm, adsorption is steep at low relative pressure, suggesting the presence of microporous structures with a large surface area. In contrast, type IV isotherms exhibit a hysteresis loop at high relative pressure, suggesting the presence of mesoporous structures and the occurrence of capillary condensation. The appearance of both isotherm types implies that the magnetic carbon composites contain both microporous and mesoporous structures. Table 1 shows the nitrogen sorption analysis results, detailing the porous textural properties of the carbon composites, including total pore volume, specific surface area (S_BET_), and both micropore and mesopore volumes.

The influence of KOH activation on porosity and the carbon material structure varies according to the quantities of KOH in the precursor solution. At KOH-to-biomass loading ratios of 0.6 and 0.8, activation created new pores and enlarged existing pores through pore widening [58]. A KOH-to-biomass ratio of 0.6 was associated with a notable increase in porosity, as the total pore volume increased to 0.70 cm^3^/g, while the S_BET_ reached 1238 m^2^/g. Further increasing the KOH-to-biomass ratio to 0.8, however, may have produced excessive activation, indicated by aggressive etching that weakened the carbon structure, causing the collapse of walls and pores [59]. Accordingly, the total pore volume of MCC-0.8 dropped to 0.46 cm^3^/g, while the S_BET_ declined to 874 m^2^/g, confirming the findings of earlier studies [47,60]. The distribution of pore sizes provided further evidence supporting these changes in the structure (Figure S1). 

Significantly, the quantities of KOH employed in this study were far smaller than those used in earlier works, yet the activation was still effective. KOH is normally applied at higher ratios, frequently exceeding 1:2 or 1:3 (biomass/KOH) [61] to ensure adequate pore formation. In contrast, the present study achieved successful outcomes with a biomass-to-KOH ratio of just 1:0.6. The ability of KOH to deliver pore activation at such a low ratio suggests that iron ions may also have an important role to play in pore formation. Additionally, the optimal KOH amount helped minimize aggressive etching, which could otherwise weaken the carbon structure and lead to pore and wall collapse. In a broader context, the use of KOH at this low ratio also reduces expenses and presents a lower environmental threat, thereby enhancing sustainability.

The functional groups of all thermally processed magnetic carbon composites were examined by FTIR spectroscopy. The FTIR spectra (Figure S2) revealed key absorption bands corresponding to functional groups of the carbon matrix as well as embedded magnetic particles. The peak at around 1746 cm^−1^ indicated the C=O stretching vibration of carbonyl or carboxylate groups. The asymmetric and symmetric stretching vibrations of carboxylate were detected at 1530 cm^−1^ and 1384 cm^−1^, respectively. Furthermore, C–O stretching in ether or carboxylate functional groups was indicated by peaks at 1180 cm^−1^ and 1077 cm^−1^, revealing the presence of oxygen-containing functional groups on the carbon surface. The CO_2_ peak at 2370 cm^−1^ was due to adsorbed carbon dioxide in the porous carbon matrix, as described in earlier studies [62]. Clear peaks located at 582 cm^−1^ and 633 cm^−1^ corresponded to the stretching vibrations of Fe–O and demonstrated the presence of the Fe₃O₄ phase within the composite, especially in the case of MCC and MCC-0.4, where Fe_3_O_4_ was the primary phase. However, when Fe^0^ was the dominant phase, the corresponding peaks in the spectra of MCC-0.6 and MCC-0.8 appeared with lower intensity. These results were consistent with the XRD analysis, thereby confirming the observed phase transitions.

Given that adsorption properties were vital for evaluating the potential applications of the proposed material, it was imperative to perform preliminary adsorption tests to measure the binding capacity of each composite adsorbent. As shown in Table 1, MCC and MCC-0.4 exhibited relatively low adsorption capacities, reaching 209.58 ± 0.64 mg/g and 254.14 ± 0.34 mg/g, respectively. This performance corresponds to their more limited pore structures, as reflected by their moderate surface areas (435 and 451 m^2^/g) and low total pore volumes (0.14 and 0.22 cm^3^/g). In comparison, MCC-0.8 achieved a higher adsorption capacity of 705.84 ± 1.26 mg/g, supported by a relatively high surface area (874 m^2^/g) and total pore volume (0.46 cm^3^/g). However, its lower micropore volume (0.27 cm^3^/g) and mesopore volume (0.18 cm^3^/g) compared to MCC-0.6 indicate a less optimal pore structure, which may have limited the available adsorption sites and, consequently, its overall adsorption performance. Notably, MCC-0.6 demonstrated the highest adsorption capacity, reaching 872.19 ± 0.28 mg/g, which can be attributed to its favorable textural properties. It possesses a large specific surface area (1238 m^2^/g) and a high total pore volume (0.70 cm^3^/g), with a well-balanced distribution between micropores (0.39 cm^3^/g) and mesopores (0.31 cm^3^/g). The micropores contribute significantly by providing confined spaces and closely spaced pore walls that promote strong pore-filling adsorption of tetracycline (TC), while mesopores facilitate multilayer adsorption by offering sufficient space for the accumulation of TC molecules. This complementary microporous–mesoporous structure likely accounts for the superior adsorption performance of MCC-0.6. Therefore, MCC-0.6 was selected as the most promising candidate for further investigation. Moreover, the composite exhibited a strong saturation magnetization value of 26.04 emu/g, which is beneficial for practical applications, as it allows efficient magnetic separation from aqueous solutions after the adsorption process, making it not only highly effective but also easy to recover and reuse.

**Figure 2:**
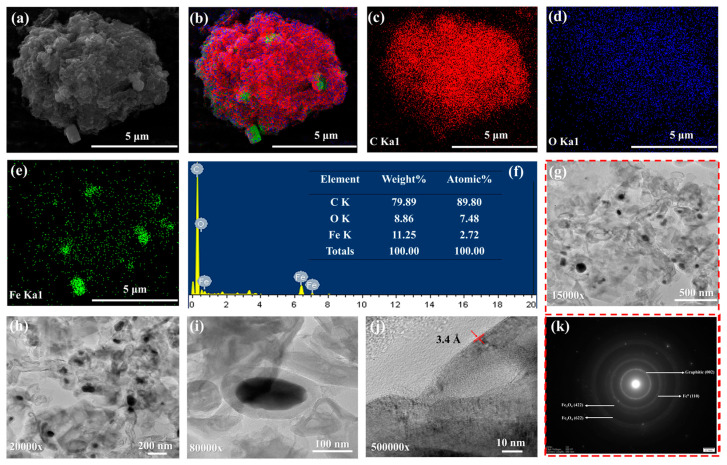
The SEM image of MCC-0.6 at 15,000× magnification (**a**), EDX elemental mapping of the composite showing carbon, iron, and oxygen (**b**), EDX mapping of carbon (**c**), EDX mapping of oxygen (**d**), EDX mapping of iron (**e**), EDX spectrum of MCC-0.6 (**f**), TEM images of MCC-0.6 at different magnifications (**g**–**j**), and selected area electron diffraction (SAED) pattern from (**g**), indicating the presence of Fe_3_O_4_, Fe^0^, and graphitic carbon (**k**).

The morphology of MCC-0.6 was observed by SEM-EDX to identify the elemental composition and confirm the existence of magnetic particles and carbon components. Figure 2a presents an SEM image showing the rough surface of the magnetic carbon composite. Embedded particles are visible, distributed widely across the carbon matrix. These embedded areas are indicative of iron-based phases. An analysis of SEM micrographs using ImageJ software (version 1.54g) revealed that the composite particles ranged in size from 2.2 μm to 18.8 μm, reflecting a relatively broad distribution likely caused by the physical grinding process. The corresponding size distribution histogram is shown in Figure S3a. Figure 2f presents the EDX spectrum of the composite with its elemental composition. The peaks in the spectrum correspond to carbon, oxygen, and iron, providing evidence of a carbon framework containing magnetic iron phases. The clear carbon signal confirmed that the principal component of the composite was carbonaceous, while the evidence of iron confirmed the incorporation of magnetic particles. The oxygen signal originated from oxygen-containing functional groups on the carbonaceous material as well as from the formed iron oxides. The spectral data were supported by color-coded elemental mapping (Figure 2b–e), which shows the distribution of the main elements in the composite. The mapping revealed that while carbon was uniformly distributed across the surface of the composite, iron was localized in specific regions where the magnetic particles were embedded.

TEM provided high-resolution evidence concerning the crystal structure, morphology, and particle distribution of the magnetic composite, along with details of the interface between the carbon matrix and iron-based particles. Figure 2g–i show TEM images of the carbon matrix containing embedded nanoparticles, confirming that magnetic components were effectively integrated. The nanoparticles in the matrix do not appear to be aggregated, indicating their stable interaction with the surrounding carbon framework. Furthermore, a d-spacing value of 3.4 Å was recorded for MCC-0.6 (Figure 2j), which is characteristic of (002) plane of graphitic carbon [63]. This result aligns with the XRD analysis, where a graphitic peak was clearly visible in the sample. The elemental composition of MCC-0.6 was revealed by TEM/EDX analysis. Peaks corresponding to carbon, iron, and oxygen revealed the existence of a carbon matrix along with iron-based magnetic phases (Figure S4). The carbon phase was evenly distributed across the matrix according to the elemental mapping, while iron was localized in the magnetic nanoparticles. These results matched those obtained from SEM/EDX. The iron particle phases were more clearly apparent in the electron diffraction patterns shown in Figure 2k. Rings observed in the selected area electron diffraction (SAED) pattern correspond to the (110) plane of Fe^0^ [64], the (422) and (622) planes of Fe_3_O_4_ [65], and the (002) graphitic plane of carbon [66]. These patterns are consistent with the results from the XRD analysis, confirming the crystalline nature of all three phases.

The TEM analysis revealed that the magnetic particle sizes ranged from 62 nm to 630 nm, reflecting a broad size distribution (Figure S3b). This can be attributed to the in situ synthesis method involving a complex biomass matrix like sugarcane bagasse. As a naturally heterogeneous material, bagasse presents uneven surface chemistry, porosity, and functional group distribution, which leads to non-uniform nucleation and growth of magnetic particles [67]. This variation means that in some regions, many small magnetic particles begin to form at once, while in other regions—where fewer particles initially form—those individual particles have more space and resources to grow larger over time. Moreover, the absence of size-controlling agents during synthesis and the possible aggregation of nanoparticles inside the porous structure also promote a wider distribution. These factors collectively result in a broader size distribution compared to nanoparticles synthesized separately under more controlled conditions. 

An XPS analysis of MCC-0.6 was conducted to identify the surface chemistry and elemental states of the adsorbent material. The XPS survey spectrum (Figure S5a) identified carbon, iron, and oxygen on the composite surface, confirming the coexistence of the carbon matrix and iron-based magnetic phases. A summary of the binding energies, peak assignments, and elemental compositions of MCC-0.6 from the XPS analysis is provided in the Table S1. In the high-resolution C 1s spectrum (Figure S5b), six visible peaks were observed at binding energies of 283.753, 284.523, 285.360, 286.253, 287.145, and 288.246 eV, corresponding, respectively, to sp^2^ C=C (defect), sp^2^ C=C, C–C, C–O, C=O, and O=C–O [27,68]. The greatest level of intensity was observed for sp^2^ graphitic carbon. This finding was consistent with the XRD peak observed at 26.23°, indicating the formation of graphitic carbon. Furthermore, in the high-resolution O 1s spectrum (Figure S5c), peaks at binding energies of 530.102, 531.024, 532.012, and 533.084 eV were observed, which correspond respectively to Fe_3_O_4_, C=O, O–C=O, and C–O–C [69,70]. These peaks indicated the presence of both iron oxide and oxygen-containing groups on the carbon material. The Fe 2p spectrum (Figure S5d) exhibited peaks around 707.287 and 720.542 eV, indicative of Fe^0^. Meanwhile, peaks at 710.603 and 712.444 eV indicated Fe 2p_3/2_, and peaks at 723.607 and 725.878 eV were attributed to the Fe 2p_1/2_ of Fe_3_O_4_ [27,49]. The XPS, SEM/EDX, and FTIR studies provided deeper insight into the magnetic carbon surface composition and chemical states of the proposed magnetic carbon composite adsorbent.

### 2.2. Adsorption of Tetracycline

**Figure 3:**
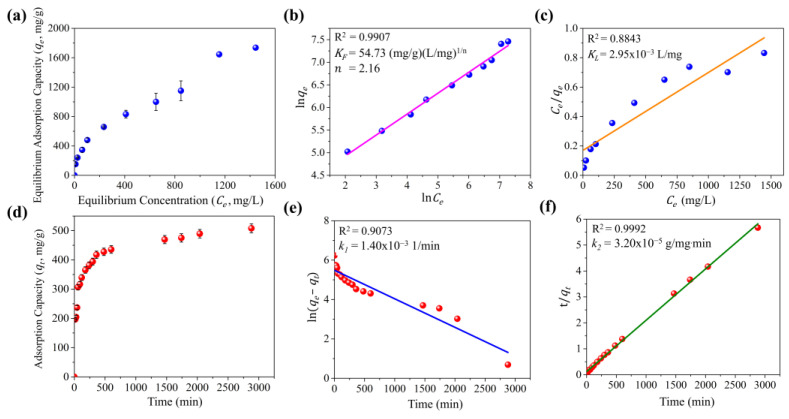
Studies of tetracycline adsorption on MCC-0.6: adsorption isotherm (**a**), linear fitting of the Freundlich isotherm model (**b**), linear fitting of the Langmuir isotherm model (**c**), adsorption kinetics (**d**), linear fitting of the pseudo-first-order kinetic model (**e**), and linear fitting of the pseudo-second-order kinetic model (**f**).

Figure 3a shows the adsorption isotherm of TC on MCC-0.6. The Langmuir and Freundlich models were employed to assess the adsorption of TC on the composite surface. The Langmuir isotherm assumes monolayer adsorption on a homogeneous surface, in which a finite number of identical sites are available. In contrast, the Freundlich isotherm empirically models adsorption on a heterogeneous surface with sites of varying affinities. The linear fitting curves are shown in Figure 3b,c, and the corresponding fitting parameters are summarized in Table S2. The results revealed that the Freundlich model (R^2^ = 0.9907) provided a better fit than the Langmuir model (R^2^ = 0.8843), indicating that TC adsorption on MCC-0.6 occurred through multilayer adsorption on a heterogeneous surface. The Freundlich constant ($K_{F}$) value was 54.73, while the empirical constant (n) was 2.16, demonstrating a high degree of adsorption with a favorable process [71]. Based on the isotherm data, MCC-0.6 exhibited a maximum adsorption capacity of 1736.93 mg/g at the highest tetracycline concentration tested (1500 mg/L).

The pseudo-first-order and pseudo-second-order models were employed to evaluate the kinetics of TC adsorption on MCC-0.6 (Figure 3d). The pseudo-first-order model assumes that adsorption sites are occupied at a rate directly proportional to the number of unoccupied sites, whereas the pseudo-second-order model assumes that adsorption is governed by the availability of adsorption sites and the interaction between TC and the adsorbent surface. The fitting results are presented in Figure 3e,f, with the associated parameters listed in Table S2. The findings indicated a better fit for the pseudo-second-order model, which exhibited a higher correlation coefficient (R^2^) than the pseudo-first-order model. This suggests that adsorption onto MCC-0.6 is predominantly influenced by surface interactions and site availability.

While the kinetic and isotherm models provided a good fit for the adsorption of TC on MCC-0.6 under controlled laboratory conditions, it is important to note that these models are based on idealized assumptions. In practical applications, factors such as fluctuating concentrations of TC, the presence of other competing pollutants, and the complex dynamics of adsorption in real systems may influence the efficiency and applicability of these models. Additionally, while the Freundlich model indicated favorable adsorption conditions, the heterogeneous nature of the surface may lead to less predictable performance in diverse environmental conditions. These aspects should be considered when extrapolating laboratory results to practical applications in environmental settings or large-scale operations.

**Figure 4:**
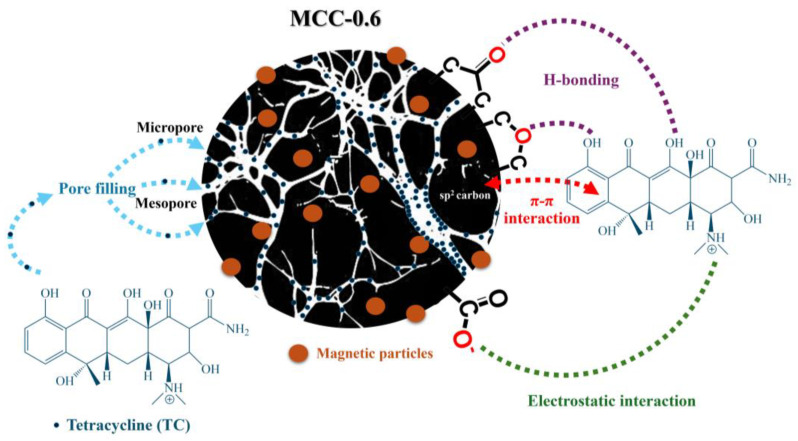
The possible mechanism of tetracycline adsorption on the MCC-0.6 adsorbent.

Figure 4 shows the proposed mechanism for the adsorption of TC on MCC-0.6. The process was influenced by both pore properties and surface chemistry. The functional groups on the carbon surface could interact with TC molecules, leading to the adhesion of TC to the surface of the adsorbent. These functional groups offered differing affinities for TC, resulting in heterogeneous adsorption. The presence of sp^2^-hybridized C=C bonds on MCC-0.6, as confirmed by an XPS analysis, indicated the importance of π–π interactions between the adsorbent surface and the aromatic rings of TC molecules. This mechanism has previously been described for carbon materials with graphitic structures [72]. Additionally, oxygen-containing functional groups, such as ethers and carbonyls, can form hydrogen bonds with tetracycline molecules, while carboxylate groups may engage in electrostatic interactions. These interactions further enhance the adsorption process, complementing the primary π–π interactions. Further insights into the adsorption mechanism were obtained through the analysis of FTIR spectral changes following TC adsorption. Shifts and intensity variations in the C=O/COO^−^ stretching band at 1746 cm^−1^, along with changes in the bands between 1300–1700 cm^−1^ and 900–1300 cm^−1^ associated with carboxylate and C–O groups, respectively, were observed (Figure S6). These spectral changes suggest the involvement of these groups in the adsorption process, likely through hydrogen bonding and electrostatic interactions [33]. The appearance of new peaks in these regions, corresponding to characteristic vibrations of TC molecules, further supports the proposed adsorption mechanism [73]. Additionally, the overlap of TC-related peaks with the original functional group signals of MCC-0.6 resulted in broader and slightly shifted spectral features, reflecting complex molecular interactions at the adsorbent surface. The porous carbon also offered a large surface area, which could accommodate an abundance of adsorption sites, thus providing greater potential for interactions to take place and increasing the adsorption capacity of the material.

The role of the pore structure in the adsorption of TC was highly significant. Micropores have strong potential for adsorption, and the close proximity of their pore walls promotes pore filling by TC molecules [34,74]. On the other hand, larger mesopores facilitate multilayer adsorption and allow for the accumulation of multiple layers of TC molecules on the pore walls [75]. The optimal pore structure, composed of both micropores and mesopores, combined with π–π interactions, hydrogen bonding, and electrostatic attraction, enabled multilayer adsorption in line with the Freundlich model, resulting in excellent adsorption capacity for tetracycline, with a maximum value of 1736.93 mg/g. 

Moreover, although Fe_3_O_4_ and Fe⁰ phases were detected in the material, SEM-EDX and TEM-EDX analyses confirmed that the iron content was relatively low (approximately 2 at%). While some interaction between tetracycline and iron phases cannot be entirely ruled out, the porous carbon framework is expected to play the predominant role in the adsorption process, with the contribution of iron species being somewhat limited.

### 2.3. Comparative Study

A comparison of this work to earlier studies on magnetic carbon adsorbents is presented in Table S3. Details cover the preparation techniques; properties of the materials, including total pore volume, S_BET_, and magnetization; and also the overall adsorption performance. Adsorbent materials were selected based on their adsorption capabilities, with priority given to those that demonstrated the best TC adsorption performance. The magnetic carbon produced in this study outperformed other adsorbents due to its high total pore volume and S_BET_, resulting from effective pore activation and its surface functionalities that enhanced interactions with TC molecules. While the saturation magnetization was not as high as that of some alternatives, it was still sufficient for magnetic separation, as an external magnet could easily attract the composite. Moreover, the efficiency gained from the one-step in situ integration of carbonization, activation, and magnetization offers significant cost and time advantages. Additionally, the use of iron waste rather than commercial iron salts contributes to further cost savings. Although the literature includes studies that have employed similar in situ processes as well as those that have separated the carbonization, activation, and magnetization stages, all have relied on more expensive commercial iron salts. The proposed approach not only reduces material costs but also adds value to waste, showcasing a practical application of iron scrap in the production of highly efficient magnetic adsorbents.

### 2.4. Reusability of the Adsorbent

**Figure 5:**
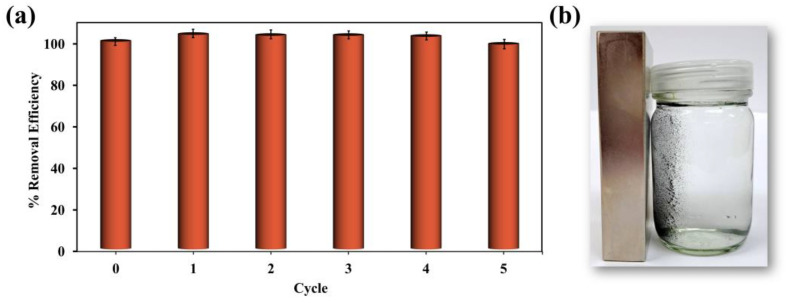
The reusability of the MCC-0.6 adsorbent for tetracycline (**a**) and a photograph showing the magnetic separation of the MCC-0.6 adsorbent after reuse (**b**).

It is vital that adsorbents can be reused, as this significantly reduces material costs and increases profitability in large-scale operations. Reusability can be assessed through a series of trials in which the adsorbent is repeatedly subjected to cycles of adsorption and desorption. An ethanol solution was used for the desorption phase. TC is moderately soluble in water, and while ethanol does not significantly enhance TC solubility, it helps weaken the surface interactions between TC molecules and the adsorbent, including π–π interactions and hydrogen bonding. This disruption promotes more efficient desorption. Following each cycle, the adsorbed TC was quantified to calculate the removal efficiency. The percent removal of the first cycle was considered 100%, serving as a baseline for comparison with subsequent cycles (Figure 5a). The percent removal remained relatively stable over five cycles, exhibiting only minor fluctuations, indicating consistent performance throughout the process.

To assess any physical or structural changes to the adsorbent after the adsorption–desorption cycle, the morphology of the material after TC adsorption and after subsequent desorption was examined by SEM (Figure S7). No obvious morphological changes were observed after these processes, indicating that the material retained its structural integrity. Additionally, SEM-EDX mapping and spectra were analyzed to monitor elemental changes. After TC adsorption, EDX mapping revealed a homogeneous distribution of nitrogen (N) on the particle surface (Figure S7c), corresponding to the presence of adsorbed TC molecules. This observation was further confirmed by the EDX spectrum (Figure S7d), where a noticeable N peak appeared. The detailed atomic percentages before adsorption, after TC adsorption, and after desorption are presented in Figure S7b,d,f. As TC was loaded, the N and O contents increased due to TC’s molecular composition. It is also worth noting that the Fe signal decreased after TC adsorption, likely due to the physical shielding of the iron-containing particles by the adsorbed TC layers. A decrease in C content was observed, which can be attributed to the normalization effect caused by the increased N and O contents. After desorption, the nitrogen signal disappeared, and the overall elemental composition returned to levels similar to the original material, suggesting successful TC desorption. These results reveal that the adsorbent retained its physical morphology and structural stability after the adsorption–desorption cycle. Additionally, the magnetic qualities of the composite remained intact after multiple regeneration cycles. The material continued to respond promptly to an external magnet after each cycle (Figure 5b), reinforcing its practical applicability for repeated use.

### 2.5. Adsorbent Application to a Real Water Resource

To provide a practical evaluation of the adsorbent, a sample of water was obtained from a source in Songkhla Province, southern Thailand. TC was added to the water to simulate contamination, reaching a concentration of 50 mg/L in a total volume of 20 mL, after which the water was treated with MCC-0.6. It was found that the magnetic carbon material was able to adsorb the TC with an efficiency close to 100%. This result suggests that MCC-0.6 could serve as a suitable adsorbent capable of removing TC from real-world water sources. In addition, no iron leaching occurred during the process, indicating that the synthesized magnetic carbon adsorbent exhibited good stability.

## 3. Materials and Methods

### 3.1. Materials

Iron scrap, obtained from Songkhla Province in southern Thailand, acted as the magnetic precursor. Potassium hydroxide (97.0%) and hydrochloric acid (37%) were supplied by J.T. Baker. The carbon precursor was sugarcane bagasse (*Saccharum officinarum* L.), obtained from Eastern Sugar and Cane Public Co. Ltd. (Sa Kaeo, Thailand). The bagasse comprised approximately 40–50% cellulose, 25–35% hemicellulose, and 15–25% lignin, along with trace amounts of minerals. Tokyo Chemical Industry Co., Ltd. (Tokyo, Japan) supplied the tetracycline hydrochloride (>98.0%). Deionized (DI) water was used in all experiments, and the chemicals employed were all of analytical reagent grade. The water samples used in the experiments were collected from a water source in Songkhla Province.

### 3.2. Preparation of Iron Scrap Solution

The iron scrap was washed to remove dust and ground into a fine powder in a 500 g multifunction disintegrator. In 50 mL of 12 M hydrochloric acid, 2 g of the iron scrap powder was dissolved to produce a solution, which was then diluted with DI water to a volume of 200 mL, giving an iron ion solution of 2000 mg/L. The concentration of the iron scrap stock solution was measured using an inductively coupled plasma optical emission spectrometer (ICP-OES, Avio 500, PerkinElmer, Waltham, MA, USA).

### 3.3. Preparation of Magnetic Carbon Materials

To prepare the sugarcane bagasse, dirt, contaminants, and any residual sugar were eliminated by washing the bagasse repeatedly with DI water. The clean bagasse was then dried at 90 °C for 48 h. The dried bagasse was milled and sieved to produce particles whose 100-mesh size was equivalent to approximately 150 µm. For the synthesis of magnetic carbon, 2.5 g of milled bagasse was mixed with 25 mL of the iron ion solution at room temperature and stirred for 1 h at 200 rpm. The planned iron-to-bagasse weight ratio was 0.02:1. KOH, at KOH-to-biomass ratios of 0.4, 0.6, and 0.8, was then dissolved in 25 mL of DI water, and the solution was introduced into the mixture of iron ion solution and bagasse. Stirring continued at 200 rpm for 15 h at room temperature. After 15 h, the mixture was heated in an oven for a further 15 h at 90 °C to evaporate water. The reference magnetic carbon material was produced in the same way but with 25 mL of DI water without KOH. 

The dried samples were labeled MCC-x, where MCC refers to the magnetic carbon composite and x represents the KOH-to-biomass ratio. Accordingly, the synthesized samples were labeled MCC-0.4, MCC-0.6, and MCC-0.8, while the sample without KOH treatment was labeled MCC as the control. The MCC-x samples were then subjected to pyrolysis under a nitrogen atmosphere. Samples were first heated to 600 °C at a rate of 2 °C/min and held for 15 min, followed by heating to 800 °C at 5 °C/min and held for 90 min. This slow heating rate helps stabilize the material by promoting a more controlled breakdown of the biomass, minimizing the rapid release of volatile components, and reducing tar formation [76]. This gradual heating ensures that intermediate products are stabilized, allowing for the carbonization process to proceed efficiently and preserving the material’s desirable porous characteristics [77], which are essential for its adsorption capacity. The temperature was then increased to 800 °C at a faster rate of 5 °C/min and held for 90 min. This second, faster heating step could enhance the development of a more porous structure, further improving the adsorption capacity of the final material [78]. After pyrolysis, the resulting materials were ground and then rinsed with hot DI water until the pH was neutral to remove soluble inorganic residues. 

### 3.4. Material Characterization

The magnetic carbon materials were degassed at 120 °C before characterization. Nitrogen sorption analysis was carried out at 77 K to measure the surface area and porosity using a Micromeritics ASAP 2460 and 3Flex analyzer, Norcross, GA, USA. The Philips X’Pert MPD, Almelo, Netherlands instrument was used to analyze the iron phase by X-ray diffraction, while the functional groups on the materials were examined by Fourier transform infrared spectroscopy using the attenuated total reflection mode (ATR–FTIR, VERTEX 70, Bruker, Billerica, MA, USA). The carbon, oxygen, and iron species were then assessed both qualitatively and quantitatively using X-ray photoelectron spectroscopy (XPS; AXIS Ultra DLD, Kratos Analytical Ltd., Manchester, United Kingdom). Measurements of magnetic properties were taken at 298 K, using vibrating sample magnetometry (VSM, Lakeshore, Westerville, OH, USA). The surface texture and iron particle distribution of composites were investigated using scanning electron microscopy with energy dispersive X-ray spectroscopy (SEM, Quanta 450, FEI, Hillsboro, OR, USA and EDX, X-Max 80, OXFORD, Abingdon, United Kingdom). The iron particles in the porous carbon and the distribution of the various elements were measured using transmission electron microscopy with energy dispersive spectroscopy (TEM-EDS, JEOL JEM-2011, and JEM 2100 Plus, Tokyo, Japan). TC concentration was determined by using UV-Vis spectroscopy (UV-2600i, Shimadzu, Kyoto, Japan), while iron ion concentrations were determined by ICP-OES (Avio 500, Perkin Elmer, Waltham，MA，USA).

### 3.5. Adsorption Studies 

The adsorption of TC on MCC-x samples was investigated by conducting isotherm experiments in which 100 mL of TC solutions at concentrations of 25, 50, 75, 100, 150, 300, 500, 750, 1000, 1250, and 1500 mg/L were adsorbed onto 0.010 g of adsorbent. The mixtures were placed in a shaker incubator (Model TOL09-FTSH-01, SCIFINETECH, Seoul, Korea) and agitated for 48 h at 30 ± 2 °C to reach equilibrium. 

In the examination of adsorption kinetics, similar conditions were applied using a 200 mg/L solution of TC that was sampled at specific time intervals up to 48 h. At each time point in both the isotherm and kinetic experiments, an external magnet was used to separate the magnetic carbon adsorbent from the sample solution. Before and after adsorption, a UV–Vis spectrophotometer was used to measure the absorbance of sample solutions at λ = 357 nm to determine TC concentrations. Equation (1) was then used to quantify adsorbed TC:

(1) $$q_{e}=\frac{\left(C_{0}-C_{e}\right)\;}{m}V$$

where $C_{0}$ is the initial adsorbate concentration (mg/L) and $C_{e}$ indicates the equilibrium concentration, V denotes the solution volume (L), and m represents the adsorbent mass (g).

To measure the adsorption capacity of adsorbent samples, adsorption isotherm models were constructed based on the linear Langmuir and Freundlich isotherm equations, as shown in Equation (2) and Equation (3), respectively.

(2) $$\frac{C_{e}}{q_{e}}=\frac{1}{K_{L}q_{max}}+\frac{C_{e}}{q_{max}}$$

(3) $$\ln q_{e}=\frac{1}{n}\ln C_{e}+\ln K_{F}$$

in which $C_{e}$ indicates the concentration of the adsorbate at equilibrium (mg/L), while $K_{L}$ (L/mg) and $K_{F}$ ((mg/g)·(L/mg)^1/n^) are the Langmuir and Freundlich adsorption constants, respectively, and n indicates the empirical constant.

A linear pseudo-first-order model (Equation (4)) and a linear pseudo-second-order model (Equation (5)) were used to model the kinetic data:

(4) $$\ln\left(q_{e}-q_{t}\right)=\ln q_{e}-k_{1}t$$

(5) $$\frac{t}{q_{t}}=\frac{1}{{k_{2}q}_{e}}+\frac{t}{q_{e}}$$

In the models shown, the symbol $q_{e}$ (mg/g) indicates the quantity of the substance adsorbed at equilibrium, while $q_{t}$ indicates the quantity adsorbed at a given time t. Meanwhile, $k_{1}$ (1/min) and $k_{2}$ (g/mg·min) represent the rate constants of the pseudo-first-order model and pseudo-second-order, respectively. 

### 3.6. Regeneration Experiments

After adsorbing TC, the isolated magnetic carbon adsorbent, MCC-0.6, was extracted in 20 mL of a 50% (*v*/*v*) ethanol solution in a 50 mL flask while shaking at 200 rpm for 12 h at 30 °C. The eluted adsorbent was washed with DI water 3 times and then reused. Five cycles of adsorption and regeneration were completed. 

## 4. Conclusions

For applications on a large scale, it is necessary to develop a low-cost adsorbent. The preparation of the magnetic carbon material (MCC-0.6) made use of sugarcane bagasse and iron scrap through a simple in situ process that integrated carbonization, magnetization, and activation in a single stage. The resulting material was highly porous with a strong capacity to remove tetracycline from aqueous solutions. The highest measured adsorption capacity toward tetracycline reached 1736.93 mg/g, which is the highest value reported to date. The proposed adsorbent could also remove tetracycline from real-world water supplies, achieving nearly 100% efficiency. Due to its magnetic properties, the material can be easily separated and recovered, facilitating reuse. Furthermore, its production utilizes waste products, offering significant environmental sustainability benefits.

## Figures and Tables

**Table 1:** Porous textural properties of magnetic carbon composites measured by nitrogen sorption at 77 K and preliminary results of tetracycline (TC) adsorption on the composites.

Material	S_BET_ (m^2^/g)	Total Pore Volume (cm^3^/g) ^a^	Micropore Volume (cm^3^/g) ^b^	Mesopore Volume (cm^3^/g) ^c^	Adsorption Capacity of TC (mg/g) ^d^
MCC	435	0.14	0.13	0.01	209.58 ± 0.64
MCC-0.4	451	0.22	0.15	0.07	254.14 ± 0.34
MCC-0.6	1238	0.70	0.39	0.31	872.19 ± 0.28
MCC-0.8	874	0.46	0.27	0.18	705.84 ± 1.26

^a^ The total pore volume is the cumulative pore volume of all pores, calculated using the density functional theory (DFT) method. ^b^ The micropore volume is the cumulative volume of pores smaller than 2 nm, calculated using the DFT method. ^c^ The mesopore volume is the cumulative volume of pores larger than 2 nm, calculated using the DFT method. ^d^ Experimental condition: initial TC concentration = 500 mg/L, solution volume = 100 mL, adsorbent dose = 0.010 g, contact time = 48 h.

## Data Availability

The original contributions presented in this study are included in the article/Supplementary Materials. Further inquiries can be directed to the corresponding authors.

## Supplementary Materials

The following supporting information can be downloaded at https://www.mdpi.com/article/10.3390/molecules30092040/s1, Note S1: The proposed mechanism of KOH as a pore activating agent in carbon; Table S1: Peak assignments in XPS spectra (C 1s, O 1s, and Fe 2p) of MCC-0.6; Table S2: Fitted parameters obtained from linear modeling of adsorption isotherms and kinetic models; Table S3: A summary and comparison of various iron-based magnetic carbon adsorbents for the removal of tetracycline; Figure S1: Density functional theory (DFT) pore size distributions of MCC (a), MCC-0.4 (b), MCC-0.6 (c), and MCC-0.8 (d). Insets show zoomed-in areas; Figure S2: FTIR spectra of MCC, MCC-0.4, MCC-0.6, and MCC-0.8; Figure S3: The particle size distribution of the composite material obtained from an SEM analysis (a) and particle size distribution of magnetic particles obtained from TEM analysis (b); Figure S4: TEM-EDX mapping of MCC-0.6; Figure S5: XPS survey spectrum of MCC-0.6 (a) and high-resolution spectra of C 1s (b), O 1s (c), and Fe 2p (d); Figure S6: FTIR spectra of MCC-0.6, MCC-0.6-TC, and TC; Figure S7: SEM images, SEM-EDX mappings, and EDX spectra of MCC-0.6 before TC adsorption (a–b), after TC adsorption (c–d), and after desorption (e–f).

## References

[1] Mishra R.K., Mentha S.S., Misra Y., Dwivedi N. (2023). Emerging pollutants of severe environmental concern in water and wastewater: A comprehensive review on current developments and future research. Water-Energy Nexus.

[2] Amangelsin Y., Semenova Y., Dadar M., Aljofan M., Bjørklund G. (2023). The impact of tetracycline pollution on the aquatic environment and removal strategies. Antibiotics.

[3] Borghi A.A., Palma M.A. (2014). Tetracycline: Production, waste treatment and environmental impact assessment. Braz. J. Pharm. Sci..

[4] Zhang Y., Shi J., Xu Z., Chen Y., Song D. (2018). Degradation of tetracycline in a schorl/H_2_O_2_ system: Proposed mechanism and intermediates. Chemosphere.

[5] Saitoh T., Shibata K., Fujimori K., Ohtani Y. (2017). Rapid removal of tetracycline antibiotics from water by coagulation-flotation of sodium dodecyl sulfate and poly(allylamine hydrochloride) in the presence of Al(III) ions. Sep. Purif. Technol..

[6] Li X., Fan S., Jin C., Gao M., Zhao Y., Guo L., Ji J., She Z. (2022). Electrochemical degradation of tetracycline hydrochloride in sulfate solutions on boron-doped diamond electrode: The accumulation and transformation of persulfate. Chemosphere.

[7] Li X., Zhou J., Jiang S., Lin Z., Jing G., He Y., Sun Y., Yang Y., Ma S., Zhang X. (2024). Photocatalytic degradation of tetracycline hydrochloride and oxytetracycline using novel zinc-based coordination polymers constructed with mixed linkers. Polyhedron.

[8] Wang C., Lin C.-Y., Liao G.-Y. (2021). Feasibility study of tetracycline removal by ozonation equipped with an ultrafine-bubble compressor. Water.

[9] Prado N., Ochoa J., Amrane A. (2009). Biodegradation by activated sludge and toxicity of tetracycline into a semi-industrial membrane bioreactor. Bioresour. Technol..

[10] Yu F., Li Y., Han S., Ma J. (2016). Adsorptive removal of antibiotics from aqueous solution using carbon materials. Chemosphere.

[11] Gao Y., Li Y., Zhang L., Huang H., Hu J., Shah S.M., Su X. (2012). Adsorption and removal of tetracycline antibiotics from aqueous solution by graphene oxide. J. Colloid Interface Sci..

[12] Hang J., Guo Z., Zhong C., Sun A., He K., Liu X., Song H., Li J. (2024). A super magnetic porous biochar manufactured by potassium ferrate-accelerated hydrothermal carbonization for removal of tetracycline. J. Clean. Prod..

[13] Liang H., Zhu C., Wang A., Chen F. (2024). Facile preparation of NiFe_2_O_4_/biochar composite adsorbent for efficient adsorption removal of antibiotics in water. Carbon Res..

[14] Chen A., Wang N., Tian Z., Wei X., Lei C. (2023). One-step synthesis of readily recyclable poplar sawdust-based porous carbon for the adsorption of tetracycline. Ind. Crops Prod..

[15] Zhao L., He P., Li Q., Pan H., Xie T., Huang S., Cao S., Liu X. (2023). Efficiently removal of tetracycline from water by Fe_3_O_4_-sludge biochar. Water, Air, Soil Pollut..

[16] Dutta J., Mala A.A. (2020). Removal of antibiotic from the water environment by the adsorption technologies: A review. Water Sci Technol..

[17] Priya S.S., Radha K.V. (2017). A review on the adsorption studies of tetracycline onto various types of adsorbents. Chem. Eng. Commun..

[18] Mansour F., Al-Hindi M., Yahfoufi R., Ayoub G., Ahmad M. (2018). The use of activated carbon for the removal of pharmaceuticals from aqueous solutions: A review. Rev. Environ. Sci. Biotechnol.

[19] Qin Y., Chai B., Wang C., Yan J., Fan G., Song G. (2022). New insight into remarkable tetracycline removal by enhanced graphitization of hierarchical porous carbon aerogel: Performance and mechanism. Colloids Surf. A Physicochem. Eng. Asp..

[20] Zhang L., Song X., Liu X., Yang L., Pan F., Lv J. (2011). Studies on the removal of tetracycline by multi-walled carbon nanotubes. Chem. Eng. J..

[21] Minale M., Gu Z., Guadie A., Kabtamu D.M., Li Y., Wang X. (2020). Application of graphene-based materials for removal of tetracyclines using adsorption and photocatalytic-degradation: A review. J. Environ. Manag..

[22] Sanni S.O., Oluokun O., Akpotu S.O., Pholosi A., Pakade V.E. (2024). Removal of tetracycline from the aquatic environment using activated carbon: A comparative study of adsorption performance based on the activator agents. Heliyon.

[23] Vinayagam R., Quadras M., Varadavenkatesan T., Debraj D., Goveas L.C., Samanth A., Balakrishnan D., Selvaraj R. (2023). Magnetic activated carbon synthesized using rubber fig tree leaves for adsorptive removal of tetracycline from aqueous solutions. Environ. Res..

[24] Jia Z., Wu L., Zhang D., Han C., Li M., Wei R. (2022). Adsorption behaviors of magnetic carbon derived from wood tar waste for removal of methylene blue dye. Diam. Relat. Mater..

[25] Zhu X., Liu Y., Zhou C., Zhang S., Chen J. (2014). Novel and high-performance magnetic carbon composite prepared from waste hydrochar for dye removal. ACS Sustain. Chem. Eng..

[26] Liu Y., Zhu X., Qian F., Zhang S., Chen J. (2014). Magnetic activated carbon prepared from rice straw-derived hydrochar for triclosan removal. RSC Adv..

[27] Deng Y., Chen J., She A., Ni F., Chen W., Ao T., Zhang Y. (2024). A novel Fe-loaded porous hydrothermal biochar for removing tetracycline from wastewater: Performance, mechanism, and fixed-bed column. J. Environ. Chem. Eng..

[28] Huang J., Wang J., Lei S., Zhang Y., Zhang M., Hu Z., Sharaf F. (2024). Iron-loaded porous semi-coke activated carbon as a highly effective and recyclable adsorbent for tetracycline removal in wastewater. Water Air Soil Pollut..

[29] Liu Y., Huo Z., Song Z., Zhang C., Ren D., Zhong H., Jin F. (2019). Preparing a magnetic activated carbon with expired beverage as carbon source and KOH as activator. J. Taiwan Inst. Chem. Eng..

[30] Zhang Z., Wang T., Zhang H., Liu Y., Xing B. (2021). Adsorption of Pb(II) and Cd(II) by magnetic activated carbon and its mechanism. Sci. Total Environ..

[31] Yang X., Wang B., Cheng F. (2024). Adsorption performance on tetracycline by novel magnetic adsorbent derived from hydrochar of low-rank coal and sewage sludge. Sep. Purif. Technol..

[32] Fuat G., Cumali Y. (2021). Synthesis, characterization, and lead (II) sorption performance of a new magnetic separable composite: MnFe_2_O_4_@wild plants-derived biochar. J. Environ. Chem. Eng..

[33] Zou C., Wu Q., Nie F., Xu Z., Xiang S. (2024). Application of magnetic porous graphite biochar prepared through one-step modification in the adsorption of tetracycline and ciprofloxacin from aqueous solutions. Waste Biomass Valorization.

[34] Sun M., Ma Y., Yang Y., Zhu X. (2023). Effect of iron impregnation ratio on the properties and adsorption of KOH activated biochar for removal of tetracycline and heavy metals. Bioresour. Technol..

[35] Xiang Y., Zhou Y., Yao B., Sun Y., Khan E., Li W., Zeng G., Yang J., Zhou Y. (2023). Vinasse-based biochar magnetic composites: Adsorptive removal of tetracycline in aqueous solutions. Environ. Sci. Pollut. Res..

[36] Cazetta A.L., Pezoti O., Bedin K.C., Silva T.L., Paesano Junior A., Asefa T., Almeida V.C. (2016). Magnetic activated carbon derived from biomass waste by concurrent synthesis: Efficient adsorbent for toxic dyes. ACS Sustain. Chem. Eng..

[37] Zeng S., Kan E. (2022). FeCl_3_-activated biochar catalyst for heterogeneous Fenton oxidation of antibiotic sulfamethoxazole in water. Chemosphere.

[38] Zhang M., Gao B., Varnoosfaderani S., Hebard A., Yao Y., Inyang M. (2013). Preparation and characterization of a novel magnetic biochar for arsenic removal. Bioresour. Technol..

[39] Pinakana S.D., Raysoni A.U., Sayeed A., Gonzalez J.L., Temby O., Wladyka D., Sepielak K., Gupta P. (2024). Review of agricultural biomass burning and its impact on air quality in the continental United States of America. Environ. Adv..

[40] Laopaiboon P., Thani A., Leelavatcharamas V., Laopaiboon L. (2010). Acid hydrolysis of sugarcane bagasse for lactic acid production. Bioresour. Technol..

[41] Rattanachueskul N., Saning A., Kaowphong S., Chumha N., Chuenchom L. (2017). Magnetic carbon composites with a hierarchical structure for adsorption of tetracycline, prepared from sugarcane bagasse via hydrothermal carbonization coupled with simple heat treatment process. Bioresour. Technol..

[42] Canilha L., Chandel A.K., dos Santos Milessi T., Antunes F.A.F., da Costa Freitas W., das Graças Almeida Felipe M., da Silva S.S. (2012). Bioconversion of sugarcane biomass into ethanol: An overview about composition, pretreatment methods, detoxification of hydrolysates, enzymatic saccharification, and ethanol fermentation. BioMed Res. Int..

[43] Kim S., Lee S.-E., Baek S.-H., Choi U., Bae H.-J. (2023). Preparation of activated carbon from korean anthracite: Simultaneous control of ash reduction and pore development. Processes.

[44] Wang Y., Chen W., Zhao B., Wang H., Qin L., Han J. (2020). Preparation of high-performance toluene adsorbents by sugarcane bagasse carbonization combined with surface modification. RSC Adv..

[45] Hiranobe C.T., Gomes A.S., Paiva F.F.G., Tolosa G.R., Paim L.L., Dognani G., Cardim G.P., Cardim H.P., dos Santos R.J., Cabrera F.C. (2024). Sugarcane bagasse: Challenges and opportunities for waste recycling. Clean Technol..

[46] Wu J., Hou B., Wang X., Liu Z., Wang Z., Liu B., Li S., Gao H., Zhu X., Mao Y. (2022). Preparation of N,S-codoped magnetic bagasse biochar and adsorption characteristics for tetracycline. RSC Adv..

[47] Fu Y., Shen Y., Zhang Z., Ge X., Chen M. (2019). Activated bio-chars derived from rice husk via one- and two-step KOH-catalyzed pyrolysis for phenol adsorption. Sci. Total Environ..

[48] Biswas A., Patra A.K., Sarkar S., Das D., Chattopadhyay D., De S. (2020). Synthesis of highly magnetic iron oxide nanomaterials from waste iron by one-step approach. Colloids Surf. A Physicochem. Eng. Asp..

[49] Thaveemas P., Chuenchom L., Kaowphong S., Techasakul S., Saparpakorn P., Dechtrirat D. (2021). Magnetic carbon nanofiber composite adsorbent through green *in-situ* conversion of bacterial cellulose for highly efficient removal of bisphenol A. Bioresour. Technol..

[50] Xu Z., Zhou Y., Sun Z., Zhang D., Huang Y., Gu S., Chen W. (2020). Understanding reactions and pore-forming mechanisms between waste cotton woven and FeCl_3_ during the synthesis of magnetic activated carbon. Chemosphere.

[51] Thiruppathi K.P., Nataraj D. (2017). Phase transformation from α-Fe_2_O_3_ to Fe_3_O_4_ and LiFeO_2_ by the self-reduction of Fe(III) in Prussian red in the presence of alkali hydroxides: Investigation of the phase dependent morphological and magnetic properties. CrystEngComm.

[52] Thompson E., Danks A.E., Bourgeois L., Schnepp Z. (2015). Iron-catalyzed graphitization of biomass. Green Chem..

[53] Hunter R.D., Ramírez-Rico J., Schnepp Z. (2022). Iron-catalyzed graphitization for the synthesis of nanostructured graphitic carbons. J. Mater. Chem. A.

[54] Gomez-Martin A., Schnepp Z., Ramirez-Rico J. (2021). Structural evolution in iron-catalyzed graphitization of hard carbons. Chem. Mater..

[55] Glatzel S., Schnepp Z., Giordano C. (2013). From paper to structured carbon electrodes by inkjet printing. Angew. Chem. Int. Ed. Engl..

[56] Chun S.E., Whitacre J.F. (2017). Formation of micro/mesopores during chemical activation in tailor-made nongraphitic carbons. Microporous Mesoporous Mater..

[57] Nogueira G.A., Fregolente L.G., Pereira L.S., Laranja M.J., Moreira A.B., Ferreira O.P., Bisinoti M.C. (2024). Magnetic activated carbonaceous materials from sugarcane bagasse: Preparation, characterization, and hexavalent chromium removal. Mater. Today Sustain..

[58] Liu P., Sun S., Huang S., Wu Y., Li X., Wei X., Wu S. (2024). KOH activation mechanism in the preparation of Brewer’s spent grain-based activated carbons. Catalysts.

[59] Zhang J., Zhang X., Li X., Song Z., Shao J., Zhang S., Yang H., Chen H. (2024). Prediction of CO_2_ adsorption of biochar under KOH activation via machine learning. Carbon Capture Sci. Technol..

[60] Song X., Zhang Y., Chang C. (2012). Novel method for preparing activated carbons with high specific surface area from rice husk. Ind. Eng. Chem. Res..

[61] Nandi R., Jha M.K., Guchhait S.K., Sutradhar D., Yadav S. (2023). Impact of KOH activation on rice husk derived porous activated carbon for carbon capture at flue gas alike temperatures with high CO_2_/N_2_ selectivity. ACS Omega.

[62] Schott J.A., Do-Thanh C.L., Shan W., Puskar N.G., Dai S., Mahurin S.M. (2021). FTIR investigation of the interfacial properties and mechanisms of CO_2_ sorption in porous ionic liquids. Green Chem. Eng..

[63] Jabarullah N.H., Kamal A.S., Othman R. (2021). A modification of palm waste lignocellulosic materials into biographite using iron and nickel catalyst. Processes..

[64] Ivanov Y., Alfadhel A., Alnassar M., Perez J., Vazquez M., Chuvilin A., Kosel J. (2016). Tunable magnetic nanowires for biomedical and harsh environment applications. Sci. Rep..

[65] Dutta S., Sharma S., Sharma A., Sharma R. (2017). Fabrication of core–shell-structured organic–inorganic hybrid nanocatalyst for the expedient synthesis of polysubstituted oxazoles via tandem oxidative cyclization pathway. ACS Omega.

[66] Sevilla M., Salinas Martínez-de Lecea C., Valdés-Solís T., Morallón E., Fuertes A.B. (2008). Solid-phase synthesis of graphitic carbon nanostructures from iron and cobalt gluconates and their utilization as electrocatalyst supports. Phys. Chem. Chem. Phys..

[67] Reynel-Ávila H.E., Camacho-Aguilar K.I., Bonilla-Petriciolet A., Mendoza-Castillo D.I., González-Ponce H.A., Trejo-Valencia R. (2021). Engineered magnetic carbon-based adsorbents for the removal of water priority pollutants: An overview. Adsorpt. Sci. Technol..

[68] Han R., Song Y., Duan J., Ai S. (2024). A recyclable biochar with ultrahigh absorption ability for efficient removal of tetracycline hydrochloride. Colloids Surf. A Physicochem. Eng. Asp..

[69] López G.P., Castner D.G., Ratner B.D. (1991). XPS O 1s binding energies for polymers containing hydroxyl, ether, ketone and ester groups. Surf. Interface Anal..

[70] Qiu C., Jiang L., Gao Y., Sheng L. (2023). Effects of oxygen-containing functional groups on carbon materials in supercapacitors: A review. Mater. Des..

[71] Sulaiman N.S., Mohamad-Amini M.H., Danish M., Sulaiman O., Hashim R. (2021). Kinetics, thermodynamics, and isotherms of methylene blue adsorption study onto Cassava stem activated carbon. Water.

[72] Huang B., Huang D., Zheng Q., Yan C., Feng J., Gao H., Fu H., Liao Y. (2023). Enhanced adsorption capacity of tetracycline on porous graphitic biochar with an ultra-large surface area. RSC Adv..

[73] Tran D.-T., Thi-My-Trinh H., Thi-An-Hang N., Viet-Cuong D., Dao V.-D. (2022). Facile preparation of reduced graphene oxide for removing tetracycline from water: Kinetics and thermodynamics studies. Sep. Sci. Technol..

[74] Wang Y., Xu L., Wei F., Ding T., Zhang M., Zhu R. (2022). Insights into the adsorption mechanism of tetracycline on hierarchically porous carbon and the effect of nanoporous geometry. Chem. Eng. J..

[75] Bao J., Zhu Y., Yuan S., Wang F., Tang H., Bao Z., Zhou H., Chen Y. (2018). Adsorption of tetracycline with reduced graphene oxide decorated with MnFe_2_O_4_ nanoparticles. Nanoscale Res. Lett..

[76] Rangabhashiyam S., Balasubramanian P. (2019). The potential of lignocellulosic biomass precursors for biochar production: Performance, mechanism and wastewater application—A review. Ind. Crops Prod..

[77] Elyounssi K., Collard F.-X., Mateke J.N., Blin J. (2012). Improvement of charcoal yield by two-step pyrolysis on Eucalyptus wood: A thermogravimetric study. Fuel.

[78] Lee S.-H., Kim J.-H., Kim W.-S., Roh J.-S. (2022). The effect of the heating rate during carbonization on the porosity, strength, and electrical resistivity of graphite blocks using phenolic resin as a binder. Materials.

