# Supplementary material for: Single-Step Upcycling of Sugarcane Bagasse and Iron Scrap into Magnetic Carbon for High-Performance Adsorbents
Source: Molecules. 2025 May 3;30(9):2040. doi: 10.3390/molecules30092040 (PMC12073645; doi:10.3390/molecules30092040)
Supplement: Supplementary file 1 [file molecules-30-02040-s001.zip › molecules-3614454-supplementary.pdf]

Supplementary Material

Single-Step Upcycling of Sugarcane Bagasse and Iron Scrap into Magnetic Carbon for High-Performance Adsorbents

Sirinad Mahawong [1,†], Piyatida Thaveemas [2,†] , Parichart Onsri [2],
Sulawan Kaowphong [3], Waralee Watcharin [4], Supanna Techasakul [2],
Decha Dechtrirat [2,5,6,*], and Laemthong Chuenchom [1,7,*]

[1] Division of Physical Science, Faculty of Science, Prince of Songkla University, Songkhla 90112, Thailand

[2] Laboratory of Organic Synthesis, Chulabhorn Research Institute, Bangkok 10210, Thailand

[3] Department of Chemistry, Center of Excellence in Materials Science and Technology, Faculty of Science, Chiang Mai University, Chiang Mai 50200, Thailand

[4] Faculty of Biotechnology, Assumption University, Hua Mak Campus, Bangkok, 10240, Thailand

[5] Department of Materials Science, Faculty of Science, Kasetsart University, Bangkok 10900, Thailand

[6] Specialized Center of Rubber and Polymer Materials for Agriculture and Industry (RPM), Faculty of Science, Kasetsart University, Bangkok 10900, Thailand

[7] Center of Excellence for Innovation in Chemistry, Faculty of Science, Prince of Songkla University, Songkhla 90112, Thailand

† These authors contributed equally to this work.

* Corresponding author; E-mail address: fscidcd@ku.ac.th, laemthong.c@psu.ac.th

Note S1. The proposed mechanism of KOH as a pore activating agent in carbon. [1]

$6KOH + 2C$	$\rightarrow$	$2K + 3H_2 + 2K_2CO_3$	(R1)
K_2CO_3	$\rightarrow$	$K_2O + CO_2$	(R2)
$K_2CO_3 + 2C$	$\rightarrow$	$2K$ (or K_2O) $+ 3CO$	(R3)
$K_2O + C$	$\rightarrow$	$2K + CO$	(R4)

Table S1. Peak assignments in XPS spectra (C 1s, O 1s, and Fe 2p) of MCC-0.6.

Peak	Binding energy	%Area	Assign Peak
**C 1s**	283.753	5.8	sp^2 C=C (defect)
	284.523	68.6	sp^2 C=C
	285.360	14.3	C-C
	286.253	5.9	C-O
	287.145	3.5	C=O
	288.246	1.9	O=C-O
**O 1s**	530.102	19.4	Fe_3O_4
	531.024	26.9	C=O
	532.012	29.4	O-C=O
	533.084	24.3	C-O-C
**Fe 2p**	707.287	5.1	Fe^0
	710.603	17.6	Fe^{2+} in Fe_3O_4
	712.444	34.7	Fe^{3+} in Fe_3O_4
	717.613	6.6	Satellite peak
	720.542	4.2	Fe^0
	723.607	15.1	Fe^{2+} in Fe_3O_4
	725.878	15.1	Fe^{3+} in Fe_3O_4
	729.422	1.6	Satellite peak

Table S2. Fitted parameters obtained from linear modeling of adsorption isotherms and kinetics models.

Adsorption models	Parameters	
**Adsorption isotherms**		
Freundlich isotherm	K_F	54.73 (mg/g)·(L/mg)$^{1/n}$
	n	2.16
	R^2	0.9907
Langmuir isotherm	q_{max}	2000.00 (mg/g)
	K_L	2.95 x 10^{-3} (L/mg)
	R^2	0.8843
**Adsorption kinetics**		
Pseudo-first-order	k_1	1.40 x 10^{-3} (1/min)
	q_e	241.94 (mg/g)
	R^2	0.9073
Pseudo-second-order	k_2	3.20 x 10^{-5} (g/mg·min)
	q_e	500.00 (mg/g)
	R^2	0.9992

Table S3. A summary and comparison of various iron-based magnetic carbon adsorbents for the removal of tetracycline.

Adsorbent	Carbon source	Magnetic source	Preparation protocol	S_{BET} (m^2/g)	Total pore volume [a, b] / Mesopore volume [c] (cm^3/g)	Saturation magnetization (emu/g)	Adsorption capacity (mg/g)	Ref
Magnetic carbon composite (MCC-0.6)	*Sugarcane bagasse*	*Iron scrap waste*	***Simultaneous carbonization, activation, and magnetization***<br>*1) Mix 2.5 g of bagasse with 25 mL of the iron scrap solution, stirring at 200 rpm for 1 h at room temperature.*<br>*2) Add KOH and stir for 15 h at 30°C, then heat in an oven at 90°C for 15 h.*<br>*3) Heat the sample to 600°C under a N_2 atmosphere for 15 min with a heating rate of 2°C/min, followed by heating at 800°C under N_2 for 90 min with a ramp rate of 5°C/min.*<br>*4) Wash the samples thoroughly with hot DI water until pH is neutral.*	*1238*	*0.70 [a] / 0.31*	*26.04*	*1736.93*	*This work*
Iron-doped biochar (Fe@C)	Cotton	$Fe(NO_3)_3$	***Simultaneous carbonization and magnetization***<br>1) Wash cotton with NaOH, oven dry at 120°C for 24 h, and crush.<br>2) Impregnate 1 g of cotton with 40 mL of $Fe(NO_3)_3$ solution (75 g/L).<br>3) Dry the sample at 60°C for 72 h.<br>4) Carbonize the material at 850°C for 1 h with a heating rate of 20°C/min.<br>5) Wash the obtained biochar with water and vacuum dry at 60°C for 12 h.	45.95	0.079 / N/A	94.65	1492.4	[2]

Adsorbent	Carbon source	Magnetic source	Preparation protocol	S_{BET} (m^2/g)	Total pore volume [a, b] / Mesopore volume [c] (cm^3/g)	Saturation magnetization (emu/g)	Adsorption capacity (mg/g)	Ref
Magnetic porous biochar (MB300-4)	Kelp	K_2FeO_4	***Simultaneous hydrothermal carbonization and magnetization***<br>1) Rinse kelp with tap water and ultrapure water, dry at 60°C, and pulverize.<br>2) Mix 6 g of kelp powder and 6 g of K_2FeO_4 with 30 mL ultrapure water in a flange reactor.<br>3) Stir the mixture evenly, heat at 5°C/min, and hydrothermally carbonize at 300°C in a muffle furnace to produce magnetic biochar.	51.63	0.207 / N/A	56.48	1245.4	[3]
Magnetic biochar (S3C7-Fe)	Sewage sludge (SS) and low-rank coal (LC)	K_2FeO_4	***Hydrothermal carbonization***<br>1) Blend SS and LC in a specified ratio with DI water at a liquid-to-solid ratio of 10:1, stir the mixture, and transfer it to a 250 mL reactor.<br>2) Heat the reactor to the desired temperature and maintain it for the required duration, then cool the reactor to room temperature and separate the solid and liquid phases to obtain hydrochar.<br>***Magnetization***<br>3) Mix 10 g of hydrochar with 20 g of K_2FeO_4 in 100 mL of deionized water.<br>4) Stir the mixture for 6 hours and dry in an oven at 80°C overnight.<br>5) Transfer the dried mixture to a reactor and heat at 800°C for 2 hours.<br>6) Wash the solid product 3 times with deionized water and 0.1 M HCl.<br>7) Dry the washed product in an oven at 105°C to obtain the adsorbent.	82.9	0.113 / 0.1067	20.05	884.04	[4]

Adsorbent	Carbon source	Magnetic source	Preparation protocol	S_{BET} (m^2/g)	Total pore volume [a, b] / Mesopore volume [c] (cm^3/g)	Saturation magnetization (emu/g)	Adsorption capacity (mg/g)	Ref
Magnetic porous graphite biochar (MBC)	Sugarcane bagasse	$FeCl_3$	***Simultaneous carbonization, activation, and magnetization***<br>1) Mix 3.0 g sugarcane bagasse and 9.0 g $ZnCl_2$ with 50 mL of 3.0 mol/L $FeCl_3$ aqueous solution in a conical flask.<br>2) Stir the mixture in a water bath at 80°C for 48 h.<br>3) Dry the mixture by evaporation at 80°C in a blast drying oven.<br>4) Pyrolyze the solid mixture at 800°C for 1 h under N_2 atmosphere in a tube furnace (heating rate: 5°C/min).<br>5) Cool the solid to room temperature and transfer it to 100 mL of 1 mol/L HCl solution and stir for 8 h.<br>6) Wash the product multiple times with DI water, dry at 80°C, grind, and sieve through a 100-mesh to obtain the black solid powder (MBC).	654.3	0.916 / 0.8560	25.00	691.32	[5]
Fe-loaded porous hydrothermal biochars (FeKHC900)	Corncob	$FeCl_3$	***Hydrothermal carbonization and magnetization***<br>1) Dissolve 5.0 g of $FeCl_3$ in 50 mL deionized water in a 100 mL beaker.<br>2) Add 5.0 g of corncob powder and treat the suspension ultrasonically for 30 min.<br>3) Transfer the mixture to a reaction vessel and heat at 200°C for 4 h.<br>4) Cool naturally to room temperature and collect the black solid via vacuum filtration and dry at 105°C to obtain FeHC.	1487.6	0.995 [b] / 0.5317	7.91	606.52	[6]

Adsorbent	Carbon source	Magnetic source	Preparation protocol	S_{BET} (m^2/g)	Total pore volume [a, b] / Mesopore volume [c] (cm^3/g)	Saturation magnetization (emu/g)	Adsorption capacity (mg/g)	Ref
			Activation<br>5) Grind 2.0 g of FeHC with 2.0 g of $K_2C_2O_4$ for 10 min in a mortar.<br>6) Pyrolyze in a tube furnace at 900°C for 1 h (10°C/min heating rate) under 0.1 L/min N_2 flow.<br>7) Wash the product with 0.1 M HCl and DI water until pH is neutral, and dry at 105°C.<br>8) Grind through a 100-mesh sieve.					
Iron-loaded activated semi-coke (2%Fe-SAC)	Semi-coke (SC)	$Fe(NO_3)_3$	***Carbonization and activation***<br>1) Wash ash from raw SC powder with water, then dry at 378 K to constant weight.<br>2) Mix dried SC with KOH at a 1:3 mass ratio and heat in a tubular furnace to 573 K at 283 K/min for 30 min under a nitrogen flow (80 mL/min). Label the sample as SAC.<br>***Magnetization***<br>3) Mix SAC with $Fe(NO_3)_3$ solution in a three-necked flask under nitrogen atmosphere, stir for 12 h, and filter the solid.<br>4) Dry the solid in a vacuum oven at 333 K for 12 h.<br>5) Heat the dried SAC in a tube furnace under nitrogen (100 mL/min) to 573 K at 283 K/min for 30 min, then to 1073 K for 3 h.<br>6) Cool to room temperature, grind through a 60-mesh sieve, and wash with deionized water	1378.8	0.420 [b] / N/A	N/A	558.07	[7]

Adsorbent	Carbon source	Magnetic source	Preparation protocol	S_{BET} (m^2/g)	Total pore volume [a, b] / Mesopore volume [c] (cm^3/g)	Saturation magnetization (emu/g)	Adsorption capacity (mg/g)	Ref
Magnetic $NiFe_2O_4$/ biochar (NFO/BC) composites	White poplar sawdust	$Ni(NO_3)_2$ and $Fe(NO_3)_3$	***<u>Pyrolysis</u>***<br>1) Clean sawdust with DI water, dry, and heat in a muffle furnace at 700°C for 1 h.<br>2) Cool to room temperature, wash with DI water and anhydrous ethanol, and dry to obtain BC.<br>***<u>Hydrothermal carbonization and magnetization</u>***<br>3) Disperse 0.1 g BC in 50 mL DI water via sonication for 30 min.<br>4) Add 0.1 mmol $Ni(NO_3)_2$ and 0.2 mmol $Fe(NO_3)_3$ to the dispersion.<br>5) Adjust pH to ~11 by adding concentrated ammonia dropwise.<br>6) Transfer the suspension to a 100 mL PTFE-lined autoclave and heat at 180°C for 12 h.<br>7) Collect the precipitate via centrifugation and wash with DI water and ethanol three times.<br>8) Dry the product in a vacuum oven at 80°C for 12 h to obtain NFO/BC.	356.1	0.270 [b] / N/A	15.20	420.41	[8]
Magnetic biochar WS-0.3	Walnut shells	$FeCl_3$	***<u>Simultaneous carbonization, activation, and magnetization</u>***<br>1) Select RH with particle sizes between 50 and 100 mesh as raw materials.<br>2) Mix $FeCl_3$ with the biomass in mass ratios of 0.3 at room temperature.<br>3) Add the mixed samples to DI water, stir for 2 h, and dry the solution.	1228.0	N/A	N/A	405.01	[9]

Adsorbent	Carbon source	Magnetic source	Preparation protocol	S_{BET} (m²/g)	Total pore volume [a, b] / Mesopore volume [c] (cm³/g)	Saturation magnetization (emu/g)	Adsorption capacity (mg/g)	Ref
			4) Mix the dried samples with KOH at a 1:2.5 mass ratio and place in a tube furnace under N_2 atmosphere.<br>5) Heat from room temperature to 800°C at 5°C/min and hold for 1 h.<br>6) Wash the cooled samples to neutral with deionized water and dry.					
Magnetic biochar composite (V-MFB-MCs-800)	Vinasse	$FeCl_3$ and $MnCl_2$	***<u>Simultaneous carbonization, activation, and magnetization</u>***<br>1) Mix 5 g dry ground vinasse with 100 mL DI water at 200 rpm for 30 min.<br>2) Add 10.81 g $FeCl_3$ and 3.96 g $MnCl_2$ to the mixture.<br>3) Adjust pH to 10 using dropwise NaOH (5 M) and oven-dry at 60°C for 4 h with continuous mixing.<br>4) Filter, wash the solid with DI water, and oven-dry at 100°C for 24 h.<br>5) Pyrolyze the dried solid under N_2 gas (100 mL/min) at 800°C for 1 h with a heating rate of 7°C/min.<br>6) Cool under N_2 purging, wash with DI water to neutral pH, and oven-dry at 100°C for 24 h.<br>7) Grind the product to pass a 100-mesh sieve (<0.15 mm).	161.17	0.191 [b] / N/A	N/A	331	[10]
Magnetic porous carbon adsorbent (MPC)	Poplar sawdust	$FeCl_3$	***<u>Simultaneous carbonization, activation, and magnetization</u>***<br>1) Mix 1.0 g of sawdust with $KHCO_3$ and $FeCl_3$ in a 1:2:0.3 mass ratio in 60 mL of deionized water.	1002.5	0.590 / 0.3100	35.80	288.2	[11]

Adsorbent	Carbon source	Magnetic source	Preparation protocol	S_{BET} (m^2/g)	Total pore volume [a, b] / Mesopore volume [c] (cm^3/g)	Saturation magnetization (emu/g)	Adsorption capacity (mg/g)	Ref
			2) Stir the mixture at 80°C to evaporate the liquid.<br>3) Transfer the viscous residue to an oven and dry at 40°C overnight.<br>4) Grind the dried material and heat in a tube oven to 750°C at 5°C/min under a N_2 atmosphere; maintain at 750°C for 2 h.<br>5) Wash the product 3 times with 0.5 M HCL and deionized water.<br>6) Dry the washed material to obtain magnetic porous carbon (MPC).					
Magnetic sludge biochar (MBC)	Residual sludge	$FeCl_3$ and $FeCl_2$	***<u>Carbonization</u>***<br>1) Pyrolyze dried sludge powder in a tube furnace at 700ºC for 2 h under nitrogen flow (0.1 L/min) with a heating rate of 5ºC/min.<br>2) Cool the biochar to room temperature and wash with DI water until neutral.<br>3) Dry the washed biochar in an oven to obtain BC.<br>***<u>Magnetization</u>***<br>4) Add BC to a mixed solution of $FeCl_2$ and $FeCl_3$ and stir for 60 min.<br>5) Adjust pH to 10-11 by slowly adding 4M NaOH while stirring for 60 min.<br>6) Filter, dry, and sieve (80 mesh) the suspension to prepare MBC.	125.07	0.270 [b] / N/A	8.02	204.20	[12]

[a]: The total pore volume calculated using the DFT method

[b]: The total pore volume calculated using the BJH method

[c]: Mesopore volume calculated by total pore volume – micropore volume

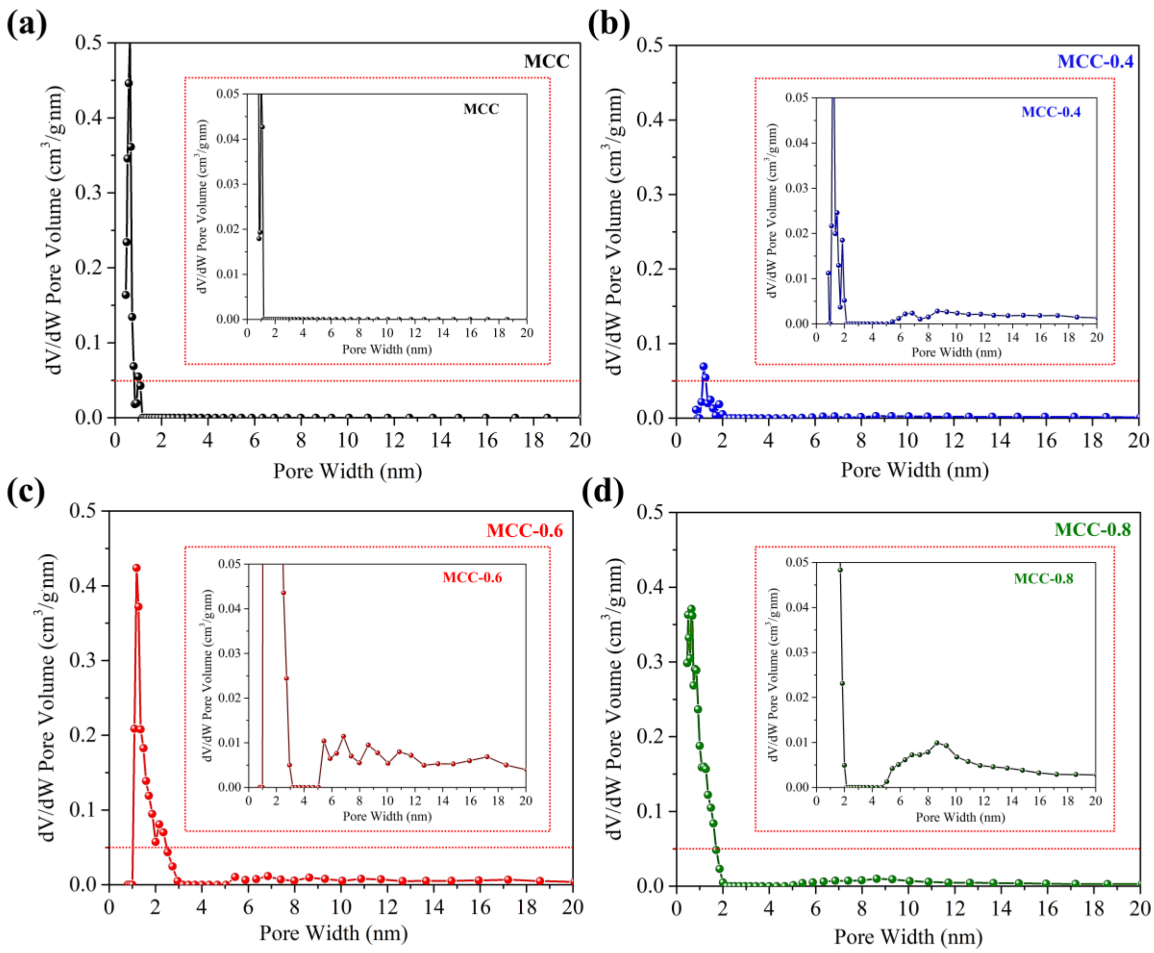

Figure S1. Density functional theory (DFT) pore size distributions of MCC (a), MCC-0.4 (b), MCC-0.6 (c), and MCC-0.8 (d). Insets show zoomed-in areas.

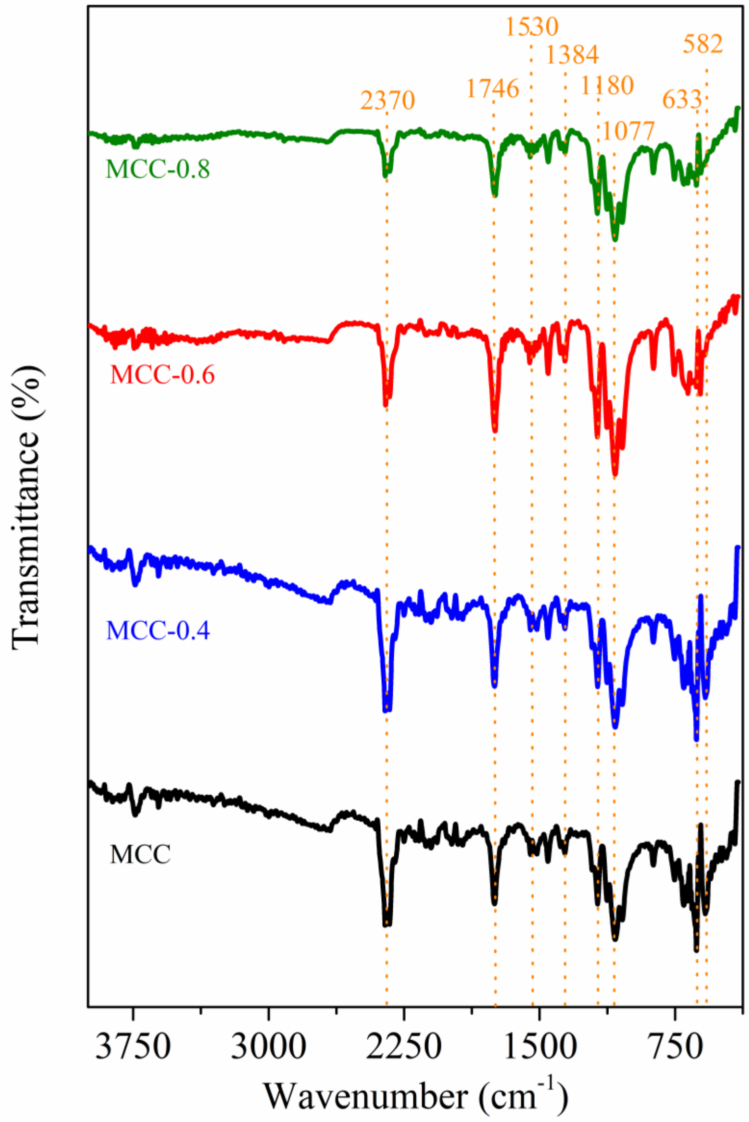

Figure S2. FTIR spectra of MCC, MCC-0.4, MCC-0.6, and MCC-0.8.

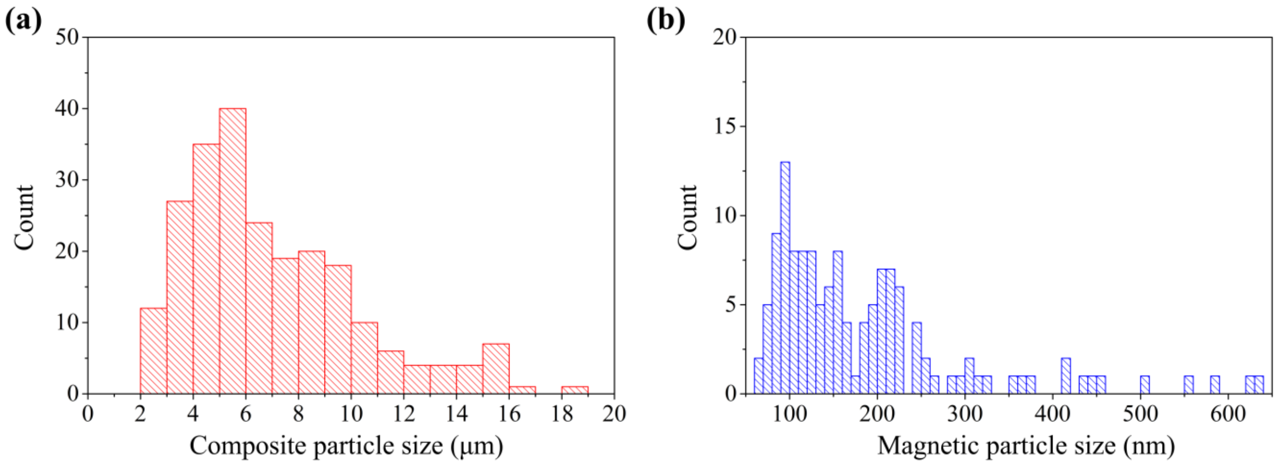

Figure S3. The particle size distribution of the composite material obtained from an SEM analysis (a) and particle size distribution of magnetic particles obtained from TEM analysis (b).

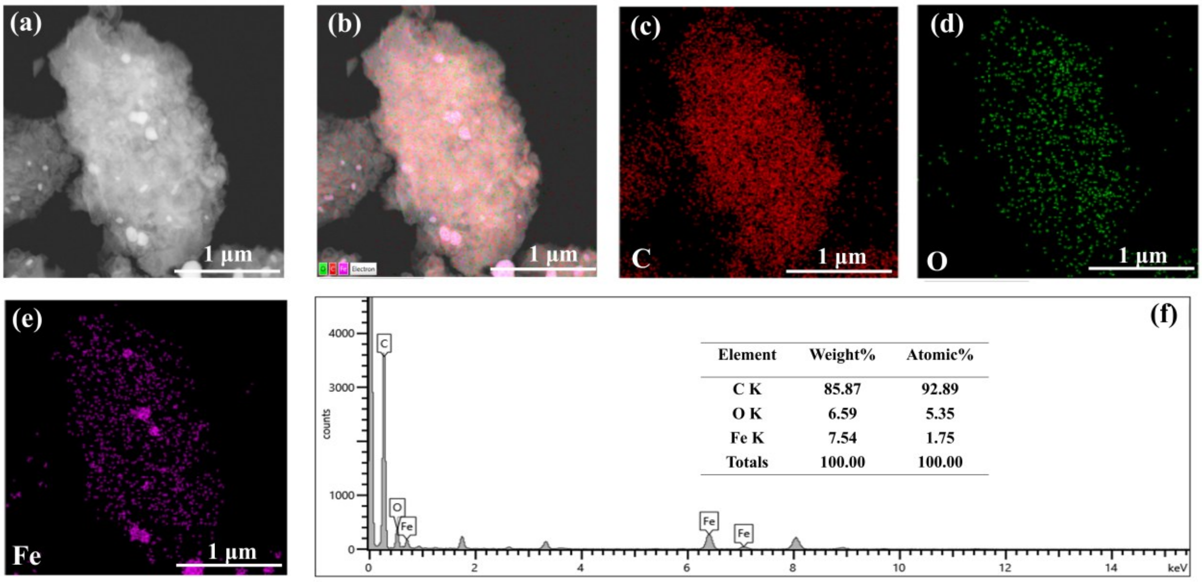

Figure S4. TEM-EDX mapping of MCC-0.6.

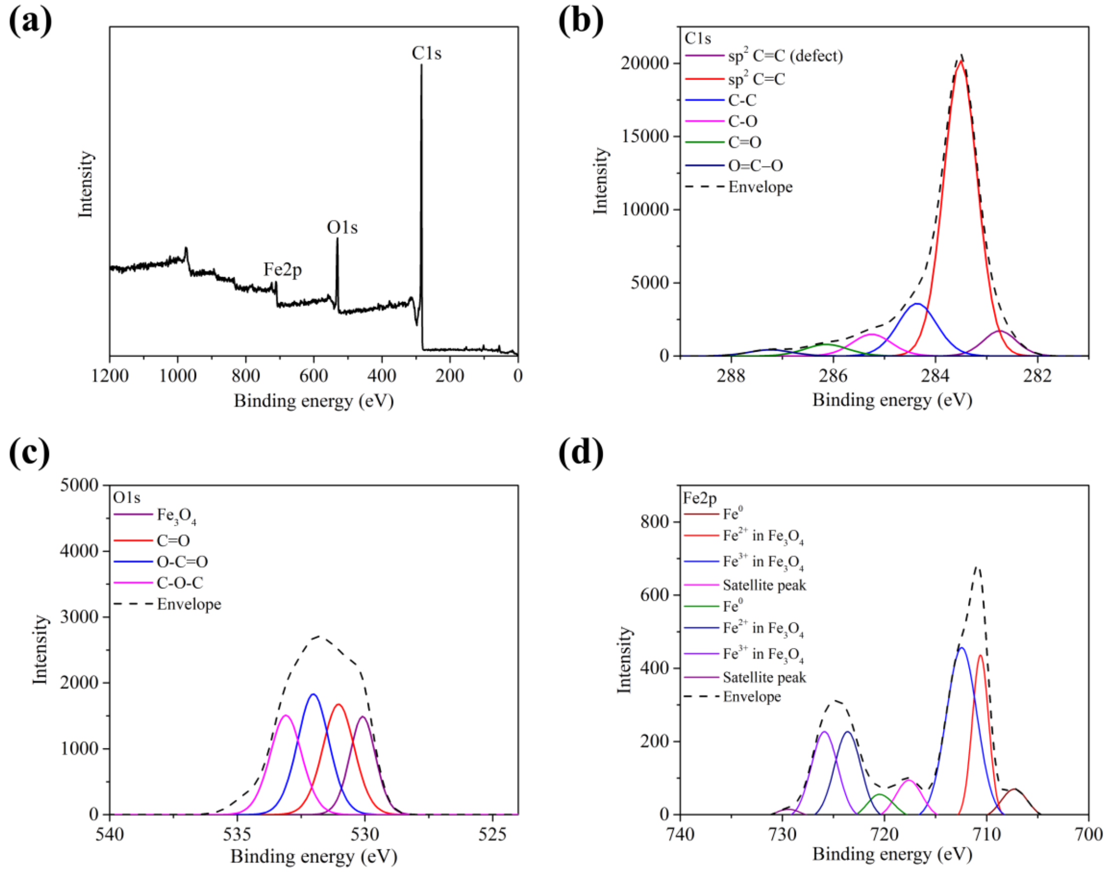

Figure S5. XPS survey spectrum of MCC-0.6 (a), and high-resolution spectra of C 1s (b), O 1s (c), and Fe 2p (d).

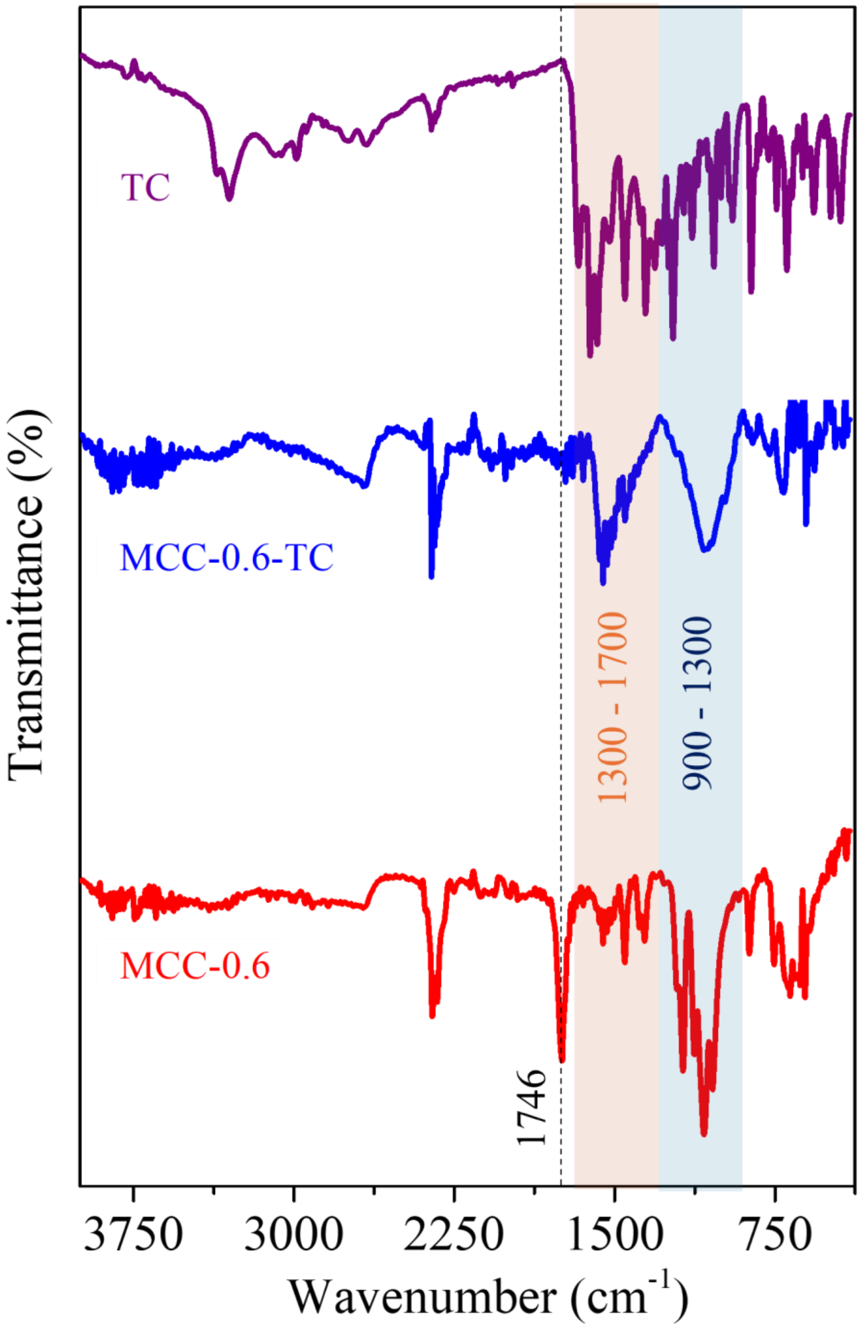

Figure S6. FTIR spectra of MCC-0.6, MCC-0.6-TC, and TC.

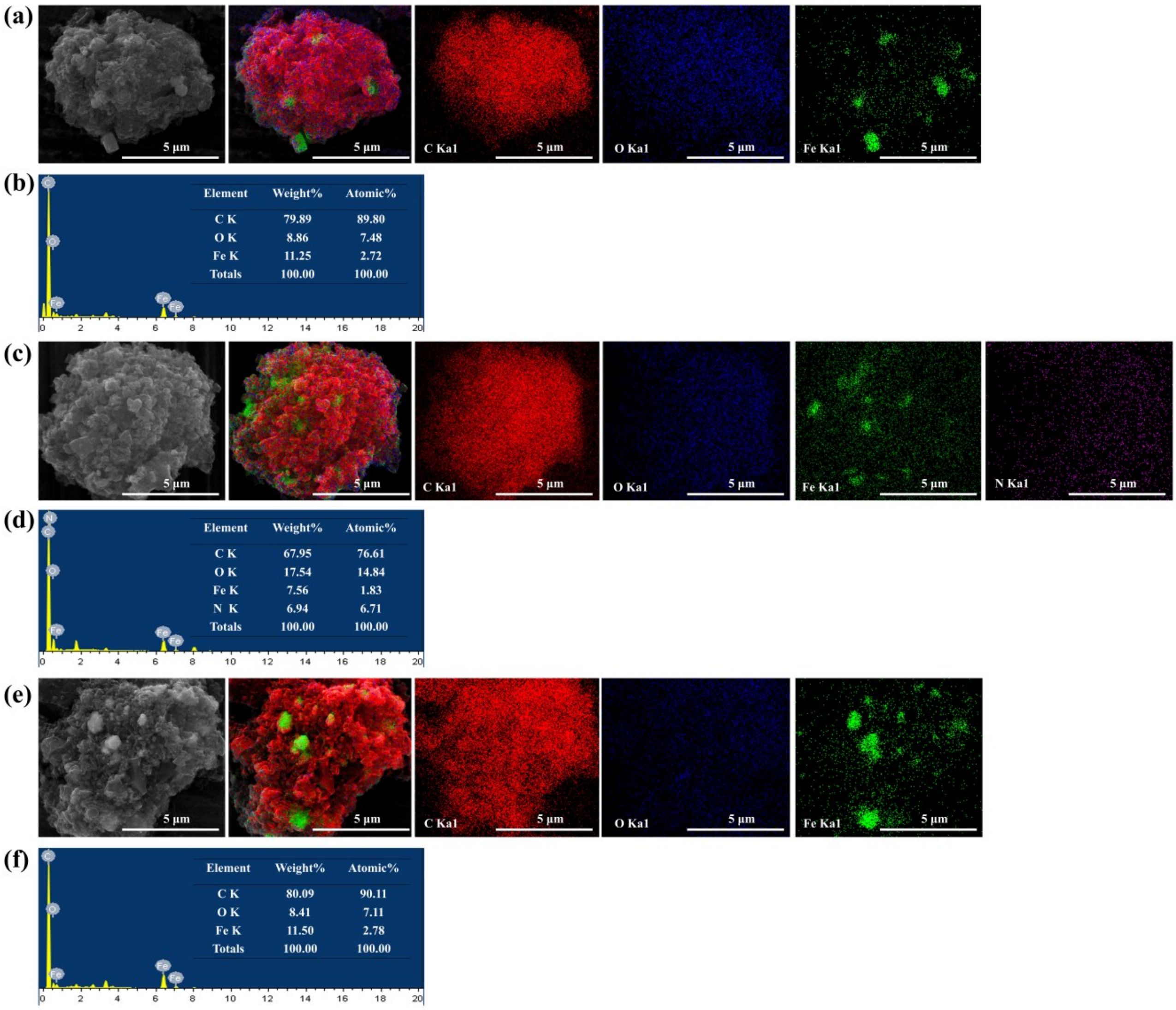

Figure S7. SEM images, SEM-EDX mappings, and EDX spectra of MCC-0.6: before TC adsorption (a–b), after TC adsorption (c–d), and after desorption (e–f).

References

(1) Fu, Y.; Shen, Y.; Zhang, Z.; Ge, X.; Chen, M. Activated bio-chars derived from rice husk via one- and two-step KOH-catalyzed pyrolysis for phenol adsorption. *Sci. Total Environ.* **2019**, *646*, 1567–1577. https://doi.org/10.1016/j.scitotenv.2018.07.423.

(2) Han, R.; Song, Y.; Duan, J.; Ai, S. A recyclable biochar with ultrahigh absorption ability for efficient removal of tetracycline hydrochloride. *Colloids Surf. A: Physicochem. Eng. Asp.* **2024**, *702*, 134974. https://doi.org/10.1016/j.colsurfa.2024.134974.

(3) Hang, J.; Guo, Z.; Zhong, C.; Sun, A.; He, K.; Liu, X.; Song, H.; Li, J. A super magnetic porous biochar manufactured by potassium ferrate-accelerated hydrothermal carbonization for removal of tetracycline. *J. Clean. Prod.* **2024**, *435*, 140470. https://doi.org/10.1016/j.jclepro.2023.140470.

(4) Yang, X.; Wang, B.; Cheng, F. Adsorption performance on tetracycline by novel magnetic adsorbent derived from hydrochar of low-rank coal and sewage sludge. *Sep. Purif. Technol.* **2024**, *330*, 125482. https://doi.org/10.1016/j.seppur.2023.125482.

(5) Zou, C.; Wu, Q.; Nie, F.; Xu, Z.; Xiang, S. Application of magnetic porous graphite biochar prepared through one-step modification in the adsorption of tetracycline and ciprofloxacin from aqueous solutions. *Waste Biomass Valorization* **2024**, *15*, 1477–1494. https://doi.org/10.1007/s12649-023-02244-y.

(6) Deng, Y.; Chen, J.; She, A.; Ni, F.; Chen, W.; Ao, T.; Zhang, Y. A novel Fe-loaded porous hydrothermal biochar for removing tetracycline from wastewater: Performance, mechanism, and fixed-bed column. *J. Environ. Chem. Eng.* **2024**, *12*, 112256. https://doi.org/10.1016/j.jece.2024.112256.

(7) Huang, J.; Wang, J.; Lei, S.; Zhang, Y.; Zhang, M.; Hu, Z.; Sharaf, F. Iron-loaded porous semi-coke activated carbon as a highly effective and recyclable adsorbent for tetracycline removal in wastewater. *Water Air Soil Pollut.* **2024**, *235*, 287. https://doi.org/10.1007/s11270-024-07105-5.

(8) Liang, H.; Zhu, C.; Wang, A.; Chen, F. Facile preparation of $NiFe_2O_4$/biochar composite adsorbent for efficient adsorption removal of antibiotics in water. *Carbon Res.* **2024**, *3*, 2. https://doi.org/10.1007/s44246-023-00094-w.

(9) Sun, M.; Ma, Y.; Yang, Y.; Zhu, X. Effect of iron impregnation ratio on the properties and adsorption of KOH activated biochar for removal of tetracycline and heavy metals. *Bioresour. Technol.* **2023**, *380*, 129081. https://doi.org/10.1016/j.biortech.2023.129081.

(10) Xiang, Y.; Zhou, Y.; Yao, B.; Sun, Y.; Khan, E.; Li, W.; Zeng, G.; Yang, J.; Zhou, Y. Vinasse-based biochar magnetic composites: adsorptive removal of tetracycline in aqueous solutions. *Environ. Sci. Pollut. Res.* **2023**, *30*, 8916–8927. https://doi.org/10.1007/s11356-022-19012-5.

(11) Chen, A.; Wang, N.; Tian, Z.; Wei, X.; Lei, C. One-step synthesis of readily recyclable poplar sawdust-based porous carbon for the adsorption of tetracycline. *Ind. Crops Prod.* **2023**, *197*, 116621. https://doi.org/10.1016/j.indcrop.2023.116621.

(12) Zhao, L.; He, P.; Li, Q.; Pan, H.; Xie, T.; Huang, S.; Cao, S.; Liu, X. Efficiently removal of tetracycline from water by Fe_3O_4-sludge biochar. *Water Air Soil Pollut.* **2023**, *235*, 38. https://doi.org/10.1007/s11270-023-06856-x.
